# An Explainable Federated Intrusion Detection Framework for SDN Using Distributed Key Generation and Threshold Homomorphic Encryption

**DOI:** 10.3390/s26175337

**Published:** 2026-08-23

**Authors:** S. M. Shamim, Yuta Kodera, Md. Arshad Ali, Yasuyuki Nogami

**Affiliations:** 1 Graduate School of Environmental, Life, Natural Science and Technology, Okayama University, Okayama 700-8530, Japan; 2 Faculty of Information and Communication Technology, Mawlana Bhashani Science and Technology University, Tangail 1902, Bangladesh; 3 Faculty of Computer Science and Engineering, Hajee Mohammad Danesh Science and Technology University, Dinajpur 5200, Bangladesh

**Keywords:** software-defined networks, federated learning, threshold homomorphic encryption, intrusion detection systems, distributed key generation, explainable artificial intelligence (XAI)

## Abstract

The rapid advancement of software-defined networking (SDN) has enhanced network programmability, centralized control, and traffic management flexibility, while also increasing exposure to sophisticated attacks targeting the control plane. Although federated learning (FL) enables collaborative intrusion detection without centralized raw data sharing, existing FL-based intrusion detection systems remain vulnerable to plaintext model update leakage, centralized cryptographic trust, limited interpretability, and insufficient validation in operational SDN environments. To address these limitations, this paper presents an explainable federated intrusion detection framework that integrates distributed key generation (DKG), CKKS-based threshold homomorphic encryption, collaborative decryption, and SHapley Additive exPlanations (SHAP). Unlike conventional HE-enabled FL systems that rely on a trusted authority or a globally shared secret key, the proposed framework removes the trusted key-generation dealer, avoids centralized custody of the complete secret key, and prevents any single client or aggregation server from independently decrypting ciphertexts using locally held key material. A gated recurrent unit (GRU)-based model is used for privacy-preserving intrusion detection, and SHAP provides global and local explanations of model decisions. The framework is further deployed in a real-time SDN testbed to evaluate the online inference pipeline following threshold-secured federated training. Computationally intensive cryptographic operations, including DKG, encrypted aggregation, and threshold decryption, are performed during offline training, while the converged global model enables low-latency inference at runtime. Experiments on the InSDN, CICDDoS2017, and CICDDoS2019 datasets with 4, 8, and 12 client federated configurations achieved detection accuracies above 99% across all datasets. The evaluation also examines encryption latency, collaborative decryption overhead, secure aggregation cost, communication complexity, and scalability. The results demonstrate that the proposed framework provides a practical balance among decentralized key management, privacy-preserving aggregation, explainability, detection performance, and real-time SDN deployment feasibility.

## 1. Introduction

Software-defined networking has emerged as a transformative networking paradigm that decouples the control and data planes, enabling centralized management, adaptive traffic control, and programmable network functions. By placing network intelligence primarily in the SDN controller, this architecture enhances scalability, flexibility, and resource efficiency across cloud, enterprise, and telecommunications infrastructures. Despite these operational advantages, the centralized nature of SDN also introduces severe security concerns [1,2]. Specifically, the SDN controller is a high-value attack target whose compromise can disrupt the entire network infrastructure through control-plane saturation, DDoS (Distributed Denial-of-Service) attacks, malicious flow manipulation, or coordinated intrusion campaigns [3,4,5].

To address these threats, machine learning (ML)- and deep learning (DL)-based IDS systems have attracted growing attention. Deep neural networks, including recurrent neural networks and gated recurrent units (GRUs), effectively learn temporal and statistical patterns from high-dimensional SDN telemetry data. Compared with traditional signature-based approaches, DL-driven IDS frameworks offer improved generalization and stronger detection capabilities against emerging network threats [6].

Despite their effectiveness, conventional IDS frameworks rely heavily on centralized training paradigms that require the collection of large volumes of network telemetry across multiple distributed SDN domains. This centralized data collection poses privacy and security risks because raw traffic flows can reveal sensitive operational details, including network topology, protocol behavior, user communication patterns, and infrastructure-specific signatures. In real-world enterprise and telecommunications environments, directly sharing raw traffic data with external aggregation servers may violate organizational privacy policies and regulatory constraints [7,8].

Federated learning has emerged as a promising solution, enabling multiple distributed clients to collaboratively train a global model without sharing private data [9]. Instead, each client trains the model locally and transmits only model updates to the aggregation server. Although FL improves privacy compared with centralized training, plaintext model updates may still expose sensitive information through model inversion and gradient reconstruction attacks [10,11]. To strengthen privacy protection in FL-based IDS systems, homomorphic encryption has been integrated into frameworks, enabling aggregation directly on encrypted model parameters [12]. In HE-enabled FL, the server aggregates ciphertexts without accessing clients’ plaintext updates. In this framework, a federated client does not represent an end host or an entity that participates directly in network forwarding. Each client represents an independently administered SDN domain, such as an enterprise, campus, data center, or edge network, that retains its own traffic telemetry. Although an SDN controller has logically centralized visibility within its local domain, it does not automatically obtain traffic data from other administrative domains. Federated learning therefore enables multiple SDN domains to develop a shared IDS model without exchanging raw network traffic.

Despite the growing adoption of HE-enabled FL, several challenges still continue to limit its practical deployment in zero-trust SDN environments. Firstly, many HE-based FL frameworks rely on centralized key generation or trusted third parties for cryptographic keys, introducing a single point of trust in the cryptographic process. If the trusted key-generation entity or the globally shared decryption capability is compromised, the confidentiality of collaborative model updates may be jeopardized. Furthermore, centralized cryptographic trust is incompatible with multi-domain SDN environments, where organizations that do not trust one another must work together and learn collaboratively without relying on any single trusted authority.

Secondly, many privacy-preserving IDS frameworks operate as black-box classifiers. Although encrypted deep learning models can achieve high detection accuracy, they often provide limited explanations for why specific traffic flows are classified as malicious. In real SDN deployments, administrators require transparent decision support before implementing mitigation actions, such as blocking traffic or modifying OpenFlow rules. Therefore, the absence of explainable AI (XAI) features reduces operational trust and restricts widespread adoption.

Thirdly, most HE-enabled FL studies are evaluated using offline datasets or simulation-based tests. While these evaluations demonstrate detection effectiveness, they do not fully address the real-time challenges of deploying IDS models in live SDN environments. Practical IDS frameworks must continuously process traffic streams, deliver low-latency inference, maintain stable throughput, and manage computational overhead effectively under both benign and malicious traffic. Consequently, the real-time feasibility of combining threshold cryptography, federated learning, and SDN-based intrusion detection has not been thoroughly examined.

Building on our previous work [13], which developed a CKKS-enabled federated intrusion detection framework for SDN environments using TenSEAL [14] and a GRU-based learning architecture, this study addresses several remaining challenges. Although the previous framework protected model updates during federated aggregation using the CKKS (Cheon–Kim–Kim–Song) homomorphic encryption scheme, its cryptographic design relied on centralized key generation and shared secret-key ownership, creating a single point of cryptographic dependency. Furthermore, it did not support threshold-secured collaborative decryption, leaving decryption capability centrally controlled and preventing the distribution of cryptographic trust among multiple participants. In addition, the previous framework did not incorporate explainability-driven IDS interpretation or real-time evaluation in practical SDN deployment environments.

**Figure 1:**
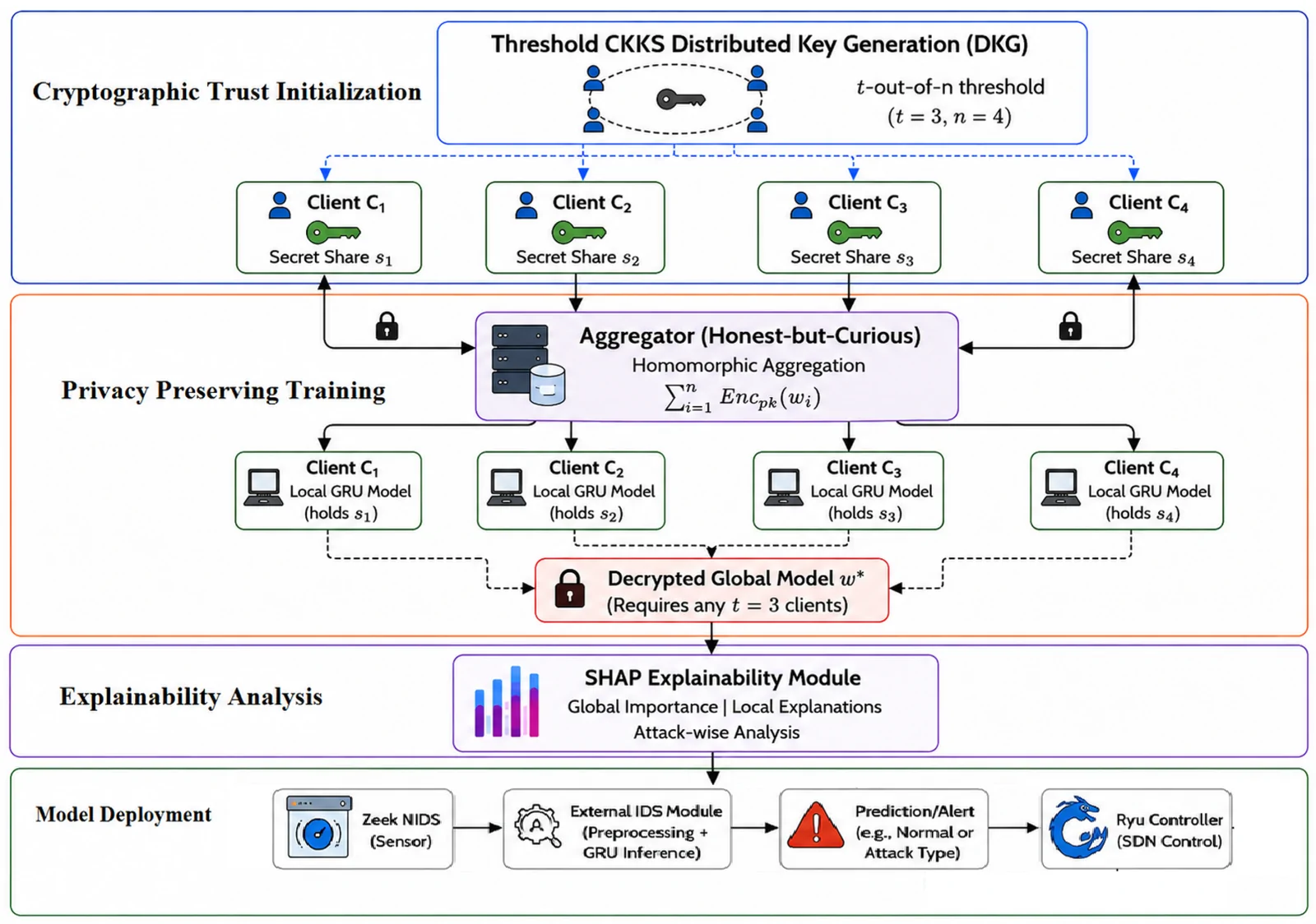
Overall workflow of the proposed federated IDS framework.

These research gaps highlight the need for a unified framework that removes reliance on a trusted key-generation dealer, distributes secret-key material among participating clients, protects federated model updates, supports explainable intrusion analysis, and enables practical SDN deployment. The proposed framework specifically addresses the limitations of key escrow and central key custody in conventional HE-enabled federated learning. In particular, the aggregation server continues to coordinate federated aggregation and collaborative decryption, while the SDN controller remains trusted for runtime network management and control-plane operation. Unlike current HE-enabled federated IDS models, which focus mainly on privacy or detection accuracy, this framework systematically evaluates the security-performance trade-off of replacing trusted-dealer key generation and centralized secret-key custody with OpenFHE-based distributed key generation and collaborative threshold decryption as illustrated in Figure 1.

The proposed threshold cryptographic design further decentralizes decryption by adopting a collaborative *t*-out-of-*n* decryption mechanism, in which at least *t* authorized clients must jointly produce valid partial decryptions before the aggregated model can be recovered. Consequently, fewer than t participating clients cannot reconstruct the plaintext aggregated model using their locally held key material. The aggregation server also cannot perform decryption independently because it holds no threshold key share. However, it can coordinate recovery of the aggregated global model when at least t clients provide valid partial decryptions. Therefore, the proposed design distributes secret-key custody and requires threshold participation rather than eliminating every form of trusted coordination.

Beyond cryptographic security, our framework also enhances the transparency and practical applicability of federated intrusion detection by enabling explainability and facilitating real-world deployment. SHAP is incorporated to provide both global and local interpretations of IDS decisions by identifying feature importance, class-specific traffic characteristics, and the model’s rationale for decisions. Furthermore, the framework is implemented in a real-time SDN testbed using Mininet [15], the Ryu controller, and Zeek NIDS (Network Intrusion Detection System) [16]. Comprehensive runtime evaluation measures detection effectiveness together with inference latency, throughput scalability, CPU utilization, and system stability under both benign and adversarial network traffic conditions.

The primary contributions of this paper are summarized as follows:

- We propose an explainable federated IDS framework for SDN that integrates OpenFHE-based distributed key generation, threshold homomorphic encryption, and collaborative threshold decryption, thereby removing the trusted key-generation dealer and centralized custody of a complete secret key.
- A t-out-of-n threshold cryptographic model is integrated into the federated learning process so that no individual client or aggregation server possesses sufficient key material to independently decrypt encrypted model updates. Recovery of the aggregated global model requires partial decryption contributions from at least t participating clients. The present framework therefore provides distributed key custody and threshold participation, while assuming that participating clients follow the prescribed collaborative-decryption procedure.
- A SHAP-based explainability framework is incorporated following threshold-secured federated training to provide global feature-importance analysis, local explanations for individual predictions, and class-wise interpretations of IDS decision behavior.
- Comprehensive experiments on the InSDN, CICDDoS2017, and CICDDoS2019 datasets systematically quantify the security–performance trade-off introduced by the proposed threshold-secured cryptographic architecture by analyzing detection performance, cryptographic overhead, and communication complexity.
- Finally, we deploy the proposed framework on a real-time SDN testbed using Mininet, the Ryu controller, and Zeek to validate the feasibility of the online inference pipeline after threshold-secured federated training.

The remainder of this paper is organized as follows. Section 2 reviews related work on federated IDS frameworks, homomorphic encryption, threshold cryptography, and explainable AI. Section 3 presents the system architecture and threat model. Section 4 describes the proposed methodology, including distributed key generation, threshold-secured federated learning, explainability analysis, and SDN deployment. Section 5 presents the experimental setup and evaluation environment. Section 6 discusses the experimental results and performance analysis. Section 7 presents the discussion, limitations, and future research directions. Finally, Section 8 concludes the paper.

## 2. Related Work

This section reviews recent studies on SDN intrusion detection, focusing on centralized deep learning, federated-based learning, homomorphic-encryption-enabled secure aggregation, explainability, and real-time deployment. The objective is to assess the extent to which existing studies address privacy preservation, decentralized cryptographic trust, interpretability, and the feasibility of practical SDN deployment.

### 2.1. Centralized Deep Learning-Based IDS for SDN

Early SDN intrusion detection frameworks primarily relied on centralized deep learning architectures trained using globally aggregated network traffic datasets. Several studies employed recurrent and convolutional architectures, including RNN, Long Short-term Memory (LSTM), Gated Recurrent Unit (GRU), Generative adversarial Networks (GAN), DBN-LSTM (Deep Belief Network-Long Short-Term Memory), and ResNet-based models for attack classification within SDN environments [17,18,19,20,21,22]. These approaches demonstrated strong classification performance for detecting DDoS attacks, botnet activities, and anomalous traffic behaviors.

In [17], the authors proposed a DRL-based IDS combining LFTS-RNN with PC-JTFOA to improve attack detection, feature selection, load balancing, and adaptive traffic management in SDN, achieving 99.85% accuracy on the NSL-KDD and WPPD datasets. In [18], a PSO-GRUGAN-IDS framework is introduced for 5G SDN environments, where PSO-optimized GAN-generated attack samples and a GRU classifier achieved 98.4% accuracy, 98.0% precision, and 98.5% recall on the InSDN dataset. Studies [19,20] developed centralized deep-learning IDS frameworks for SDN using RNN/LSTM/GRU models and a DCGAN–CenterNet–ResNet152V2–SMA pipeline, respectively, achieving high intrusion-detection performance on the InSDN and Edge-IIoT datasets while supporting DoS detection and mitigation. In [21], the authors proposed SATIDS, an improved LSTM-based two-level IDS that distinguishes benign and malicious traffic and identifies attack categories, achieving 99.73% accuracy on the InSDN dataset. In [22], an adversarial DBN–LSTM framework is developed for detecting and defending against DDoS attacks in SDN environments.

Despite their detection effectiveness, centralized learning architectures inherently require collecting and storing large-scale network telemetry on a central server. Such centralized aggregation raises serious privacy concerns because sensitive traffic information, network topology, and communication patterns are exposed to the training infrastructure. Furthermore, centralized training frameworks suffer from scalability limitations and increased vulnerability to a single point of compromise, particularly in multi-domain SDN environments where traffic data may originate from independent administrative entities.

Additionally, centralized IDS frameworks often incur substantial computational overhead due to continuous ingestion of large-scale traffic and model retraining. Although feature engineering and dataset balancing techniques can improve classification performance, these approaches do not fundamentally address the underlying privacy and trust limitations associated with centralized data aggregation.

### 2.2. Federated Learning-Based IDS for SDN

To overcome the limitations of centralized data collection, FL has emerged as a promising distributed learning paradigm for developing privacy-preserving IDS. FL enables multiple distributed SDN domains to collaboratively train a shared global model while retaining raw traffic data locally. Several recent studies have explored FL-based IDS architectures for SDN and IoT environments [23,24,25,26,27].

Recent studies show that FL can effectively improve the security of SDN networks while maintaining data privacy. For example, the authors in [23] developed an FL–based DNN (Deep Neural Network) model to identify cyberattacks in SDN-enabled aeronautical communication networks. On the NSL-KDD dataset, this FL approach achieved 96% accuracy, outperforming non-FL models at 76% and 83%, while preserving data privacy and minimizing service exposure. In [24], the authors proposed a decentralized framework that combines FL with blockchain to enhance SDN-based IoT networks. Blockchain is essential for secure, transparent, and tamper-proof logging, as well as for detecting and preventing model poisoning during training. The system also uses distributed monitoring, multi-layer encryption, and Hashgraph technology to enhance integrity and trust among users. However, this approach can lead to significant computational and communication overhead, and its performance evaluation with a private CNN-LSTM model lacks quantitative metrics such as accuracy, precision, recall, or F1-score.

The authors in [25] introduce FedLAD (Federated Evaluation of Deep Leakage Attacks and Defenses), a decentralized learning system designed to reduce DDoS attacks in large multi-controller SDN networks. By training models locally on controllers and avoiding raw data sharing, FedLAD achieves over 98% accuracy on the InSDN, CICDDoS2019, and CICDoS2017 datasets. Real-time testing shows that it maintains high detection accuracy while consuming minimal resources, highlighting its advantages over other federated learning-based methods. Another study introduced IDSFedNet [26], a lightweight, federated-learning-based IDS system designed for SDN-enabled industrial CPS. The framework uses chi-square testing and the PCC (Pearson Correlation Coefficient) for feature selection, reducing model complexity while maintaining strong performance, with reported accuracies of 90.83% on InSDN and 95.41% on Edge-IIoTset. However, the approach does not incorporate secure model aggregation and overlooks essential aspects such as model explainability and practical deployment in SDN environments.

Moreover, the study in [27] proposed a federated deep-learning–based NIDS tailored for multi-controller SDN–IoT environments, incorporating IPFS for decentralized model storage and AES encryption to secure parameter transmission. Although the system achieved an impressive 99.89% accuracy on a custom dataset, its use of standard encryption provides confidentiality only during communication. The framework does not integrate any secure aggregation mechanism and overlooks critical aspects such as model explainability and practical deployment considerations.

Although FL significantly reduces the need for centralized raw-data sharing, standard federated learning pipelines remain vulnerable to information leakage during client-server communication. In conventional FL systems, model gradients or weight updates are transmitted in plaintext to the aggregation server. Recent studies have shown that adversaries can exploit these gradients to perform model inversion, data reconstruction, and gradient-leakage attacks that reveal sensitive client-side information [28,29]. Consequently, while FL improves privacy compared with centralized learning, the lack of secure aggregation mechanisms limits its suitability for highly sensitive SDN security environments.

### 2.3. Homomorphic Encryption-Based Federated IDS

To address gradient leakage and model inversion vulnerabilities in standard federated learning, recent research has integrated homomorphic encryption (HE) into FL-based IDS frameworks. HE enables aggregation operations to be performed directly on encrypted model updates, without exposing plaintext parameters to the aggregation server, thereby improving privacy during collaborative training.

Recent advances in threshold HE and distributed key generation have further extended HE-based architectures toward decentralized cryptographic trust models. Unlike conventional HE systems that rely on a centralized, trusted dealer for secret-key generation, threshold cryptographic approaches distribute secret-key ownership among multiple participants. This ensures that no single entity can independently reconstruct the private key or decrypt aggregated ciphertexts. Prior studies have explored RLWE-based threshold encryption, distributed decryption, and multiparty cryptographic protocols for secure collaborative computation [30,31,32]. These studies demonstrate the feasibility of eliminating centralized trust assumptions while preserving secure aggregation in distributed learning environments. Moreover, recent works have highlighted the importance of threshold cryptographic architectures for mitigating single points of failure in decentralized systems and secure multiparty computation [33].

Moreover, secret-sharing-based FL frameworks have also distributed privacy-sensitive information across multiple aggregation-side entities. The authors in [34] proposed LSFL, which employs a lightweight two-server secure aggregation architecture to distribute aggregation-side trust while supporting privacy-preserving and Byzantine-robust federated learning. More recently, dual-server secure aggregation frameworks have used additive secret sharing by splitting each client model update into separate shares and transmitting them to two aggregation servers, such that neither server independently receives the complete client update [35]. In contrast, the proposed framework does not secret-share client model updates among aggregation servers. Instead, model updates are encrypted using CKKS and aggregated as ciphertexts by the aggregation server, while threshold decryption key material remains distributed among the participating federated clients. Therefore, the principal architectural difference lies in the location and function of the distributed shares: server-sharing approaches distribute model-update shares or aggregation trust across server-side entities, whereas the proposed framework distributes threshold decryption-key custody across participating client domains.

Several studies have incorporated HE-based secure aggregation into distributed IDS frameworks. For instance, ref. [36] employed homomorphic encryption alongside a 1DCNN-TransBiLSTM architecture to protect client-side gradients during federated learning. Our previous framework also protected model updates using the CKKS scheme during federated aggregation and demonstrated strong intrusion-detection capability across distributed datasets [13]. However, its cryptographic design relied on centralized key generation and globally shared secret-key ownership. Consequently, any authorized participant with access to the secret key could independently decrypt aggregated model parameters, creating a single point of cryptographic dependency.

### 2.4. Explainability and Deployment-Aware IDS Frameworks

Although secure aggregation safeguards model updates during federated training, the resulting deep-learning IDS model can still behave as a black-box decision system after training. In operational SDN environments, this opacity poses issues because automated mitigation steps—such as flow blocking or the implementation of OpenFlow rules—can affect vital network services. Consequently, privacy-preserving IDS frameworks require not only secure collaborative training but also transparent post-training decision analysis afterward.

To improve operational transparency, several recent studies have explored XAI techniques for federated learning-based IDS systems [37,38,39]. These approaches provide interpretable attack predictions and increase administrators’ trust in automated detection systems. However, many XAI-enabled IDS frameworks prioritize interpretability and do not simultaneously incorporate robust privacy-preserving mechanisms such as secure aggregation, homomorphic encryption, or decentralized trust management. Consequently, they may still be vulnerable to model leakage, gradient reconstruction, or reliance on centralized trust assumptions. Conversely, many encrypted federated IDS systems provide stronger privacy guarantees during training but offer limited human-readable explanations of attack predictions after model convergence.

In addition to interpretability, practical deployment validation remains underexplored in existing HE-enabled federated IDS studies. Most current works evaluate their frameworks using offline benchmark datasets and simulation-based experiments. However, operational SDN environments impose additional runtime constraints, including live traffic ingestion, feature extraction, online inference, controller integration, and continuous enforcement of flow rules. Therefore, a deployable SDN security framework must integrate secure offline federated training with post-training explainability and real-time inference.

### 2.5. Research Gap Summary

Table 1 provides a grouped summary of representative DL- and FL-based intrusion detection frameworks for SDN environments. Studies that share the same high-level characteristics under the selected comparison criteria are consolidated into a single row, although their individual architectures, datasets, and evaluation settings may differ. These methodological differences are discussed separately in the related-work section [17,18,19,20,21,22]. The literature indicates that existing studies generally address individual aspects of secure SDN intrusion detection, such as privacy-preserving federated learning, homomorphic-encryption-based secure aggregation, explainable artificial intelligence, or real-time deployment. However, these capabilities are typically examined in isolation rather than within a unified framework.

Based on the preceding review, several important research gaps remain unresolved. Firstly, many federated IDS architectures continue to rely on centralized cryptographic trust models, in which secret keys are generated or managed by a trusted authority, thereby introducing a single point of cryptographic failure. Secondly, although homomorphic encryption protects model updates during aggregation, most existing HE-enabled IDS frameworks do not support distributed key generation and threshold collaborative decryption, thereby limiting decentralized trust management. Thirdly, many privacy-preserving IDS models remain opaque, offering limited insight into attack predictions or feature-level decision behavior, thereby reducing operational trust and interpretability. Finally, most existing studies validate their approaches only through offline datasets or simulation-based experiments, with limited investigation of deployment feasibility in operational SDN environments.

To address these limitations, this paper proposes an explainable federated intrusion detection framework that integrates OpenFHE-based distributed key generation, threshold homomorphic encryption, collaborative threshold decryption, SHAP-based explainability, and real-time SDN deployment with Mininet, the Ryu controller, and Zeek. Unlike existing approaches that address privacy preservation, decentralized cryptographic trust, explainability, or deployment feasibility individually, the proposed framework unifies these capabilities within a single SDN intrusion detection architecture. This unified design enables privacy-preserving collaborative learning without centralized complete-secret-key custody, provides interpretable IDS decisions through SHAP-based analysis, and demonstrates practical deployment feasibility under realistic SDN operating conditions.

## 3. System Architecture and Threat Model

This section presents the overall architecture, operational entities, trust assumptions, and security boundaries of our threshold-secured federated IDS framework for SDN environments. Figure 1 illustrates the complete operational workflow of the proposed framework.

### 3.1. Architectural Overview

As illustrated in Figure 1, the framework consists of four sequential operational stages: (i) decentralized cryptographic trust initialization, (ii) privacy-preserving federated training and encrypted aggregation, (iii) explainability-driven IDS analysis, and (iv) real-time SDN deployment and online inference. These stages collectively enable collaborative intrusion detection while preserving the confidentiality of model updates and reducing dependence on centralized cryptographic trust.

In the first stage, participating federated clients establish decentralized cryptographic trust using a threshold CKKS-based distributed key generation mechanism. Unlike conventional HE-enabled FL architectures that rely on a centralized trusted authority for secret-key generation, the framework distributes secret-key ownership across participating clients. Therefore, no individual client or aggregation server can independently decrypt ciphertexts using only its locally held key material. During the second stage, each federated client trains a local GRU-based IDS model using its private traffic data partition. After local optimization, model updates are encrypted using the jointly generated public key and transmitted to the aggregation server. The server performs aggregation entirely in the ciphertext domain and therefore does not access plaintext local model updates.

In the third stage, SHAP-based explainability analysis is performed on the converged global IDS model to identify influential network-flow features and support interpretable intrusion detection. This analysis is conducted offline before real-time SDN deployment. Finally, in the fourth stage, the trained global IDS model is deployed within an SDN environment for online traffic monitoring and intrusion detection. Runtime traffic is monitored using Zeek 7.0.1, processed into flow-level features, and classified by the deployed GRU-based IDS model. Detailed deployment configuration and traffic-generation procedures are presented in the experimental setup section.

### 3.2. System Entities and Operational Components

The proposed framework consists of the following operational entities.

#### 3.2.1. Federated Clients

The set

$$C=\{C_{1},C_{2},\ldots ,C_{n}\}$$

denotes independently administered SDN domains participating in federated training. Each client maintains locally collected traffic data and independently trains the GRU-based IDS model. Clients participate in model training and threshold cryptographic operations but do not manage the networks of other clients. Network forwarding and control remain the responsibility of the local SDN controller within each administrative domain.

#### 3.2.2. Aggregation Server

The aggregation server coordinates the federated learning process by distributing the global model, collecting encrypted local updates, and performing ciphertext-domain aggregation. The server does not possess a threshold secret-key share and therefore cannot decrypt any local or aggregated ciphertext using its own key material. During the collaborative-decryption phase, however, the server collects partial decryption contributions from at least t participating clients and combines them to recover the aggregated global model. Accordingly, the proposed framework removes centralized secret-key custody and independent server-side decryption, but the aggregation server retains a coordination role in the threshold-decryption workflow.

#### 3.2.3. Explainability Analysis Module

The explainability module performs SHAP-based interpretation of the converged global IDS model after federated training. It provides global and local feature-importance analysis to improve interpretability, transparency, and analyst-level trust in intrusion-detection decisions.

#### 3.2.4. SDN Deployment Infrastructure

The SDN deployment infrastructure consists of Mininet-based topology emulation, OpenFlow-enabled virtual switches, the Ryu SDN controller, and Zeek-based traffic monitoring. The controller manages flow-level forwarding behavior, while Zeek extracts runtime flow-level telemetry for online IDS inference.

### 3.3. Threat Model and Trust Assumptions

The framework adopts a distributed cryptographic trust model for federated SDN intrusion detection. It explicitly addresses adversarial threats across the learning pipeline, cryptographic infrastructure, and communication channels. The proposed framework does not eliminate all trusted entities. Its cryptographic contribution is limited to removing the trusted key-generation dealer, avoiding centralized custody of the full secret key, and preventing any single client or aggregation server from independently decrypting ciphertexts using locally held key material. The aggregation server remains a protocol coordinator, and the SDN controller remains operationally trusted.

#### 3.3.1. Honest-but-Curious Aggregation Server

The aggregation server is assumed to be honest-but-curious. It correctly follows the federated aggregation protocol but may attempt to infer sensitive information from encrypted model updates, communication patterns, or aggregated ciphertexts. Since the server does not possess any threshold secret-key share, it cannot decrypt local or aggregated ciphertexts using its own key material. Nevertheless, the server coordinates collaborative decryption and can recover the aggregated global model after receiving partial decryption contributions from at least t clients. The security claim is therefore limited to preventing independent server-side decryption and centralized secret-key custody.

#### 3.3.2. Malicious and Colluding Clients

A subset of federated clients may be compromised or malicious. Such clients may attempt to infer sensitive training information, reconstruct secret material, decrypt aggregated ciphertexts, or collude with other participants.

#### 3.3.3. Security Bounds and Byzantine Assumptions

Let *c* denote the number of colluding clients. Under a (t,n) threshold configuration, confidentiality against client collusion is maintained as long as c<t. Therefore, the maximum number of colluding clients tolerated without enabling threshold decryption is t−1. If c≥t, the colluding clients possess sufficient threshold information to complete decryption, and confidentiality is no longer guaranteed. Model poisoning and Byzantine behavior represent a separate threat from cryptographic collusion. The framework extends the client registration, integrity verification, and Byzantine screening mechanisms introduced in our previous work [13]. Before aggregation, the server verifies client registration and ciphertext integrity and screens encrypted updates for structural anomalies. Invalid or suspicious contributions are rejected or filtered, and the training round is aborted if the number of detected suspicious clients reaches the configured Byzantine threshold.

#### 3.3.4. Collaborative-Decryption Assumption

Participating clients are assumed to follow the prescribed collaborative-decryption procedure and generate partial decryptions only for the legitimate aggregated ciphertext of the current federated round. Recovery of the authorized aggregate requires valid partial-decryption contributions from at least *t* participating clients. The current implementation does not independently verify whether a decryption request corresponds to this authorized aggregate. Therefore, the current implementation does not provide protection against arbitrary-ciphertext, repeated-decryption, or decryption-oracle requests from a malicious coordinator, and this remains future work.

#### 3.3.5. External Communication Adversaries

The communication channels between clients and the aggregation server are assumed to be observable by external adversaries. These adversaries may attempt passive eavesdropping, packet interception, or traffic analysis. Model updates are therefore transmitted only as CKKS ciphertexts, and recovery of the aggregated update requires threshold collaboration among authorized participants.

Table 2 summarizes the primary adversarial threats considered in the proposed framework and the corresponding mitigation mechanisms.

### 3.4. Cryptographic Security Objectives

The proposed framework is designed to satisfy four primary cryptographic security objectives. Firstly, it preserves the confidentiality of local model updates by encrypting all client-side model parameters before transmission. Consequently, plaintext model updates and private training data remain local to each participating client throughout the federated learning process. Secondly, the framework removes the trusted key-generation dealer and centralized storage of a complete secret key through distributed key generation. Each participating client retains only its own threshold key material, while no individual client, aggregation server, or SDN controller stores the complete secret key. This prevents any single entity from decrypting ciphertexts using locally held key material alone. However, this property does not imply that the overall SDN architecture is free from trusted operational components.

Thirdly, recovery of the aggregated global model requires threshold participation. During collaborative decryption, at least t clients generate partial decryption contributions, which the aggregation server combines to recover the plaintext aggregate. The resulting security property is distributed key custody and the absence of independent single-party decryption. The current implementation assumes that participating clients generate partial decryptions only for the protocol-defined aggregate.

Finally, the framework performs secure collaborative decryption only after encrypted aggregation is complete, allowing the recovered global model to be redistributed for the next federated learning round while the underlying secret-key shares remain distributed throughout the protocol. This design strengthens protection against centralized secret-key compromise while preserving the confidentiality of individual client updates under the stated honest-but-curious server and protocol-compliance assumptions.

## 4. Proposed Methodology

This section presents the operational methodology of the threshold-secured federated IDS framework. The proposed methodology is organized into four sequential operational phases: (i) distributed key generation, (ii) threshold-secured federated training and encrypted aggregation, (iii) explainability-driven intrusion analysis, and (iv) real-time SDN deployment and online inference.

### 4.1. Distributed Key Generation and Threshold-Share Construction

The proposed framework removes the need for a trusted key-generation dealer by combining OpenFHE multiparty CKKS key generation with a custom post-DKG threshold-share construction and a collaborative decryption procedure. All *n* registered clients participate in OpenFHE’s native multiparty key-generation procedure to establish the joint CKKS public key. This initialization stage is an *n*-party procedure and is not claimed to provide arbitrary *t*-out-of-*n* functionality. After the joint public key has been established, the local DKG secret contributions are reshared using a custom Shamir-based polynomial construction. The resulting combined client shares are used by the custom C++/Pybind decryption procedure described in Section 4.4. Therefore, the *t*-client property evaluated in this work applies to the custom post-DKG collaborative-decryption stage rather than to OpenFHE’s native multiparty DKG or native multiparty-decryption procedure [40].

#### 4.1.1. OpenFHE Multiparty Key Establishment

During initialization, client $C_{1}$ starts the sequential OpenFHE key-generation procedure by generating the first key pair. Each subsequent client $C_{i}$, i=2,…,n, executes the multiparty key-generation operation using the joined public key obtained from the preceding stage. After all *n* clients have participated, the resulting public key is denoted by pk. Each client $C_{i}$ retains its own local DKG secret contribution $s_{i}$. The corresponding implicit joint CKKS secret is represented as

(1) $$s=\sum_{i=1}^{n}s_{i}.$$

Here, $s_{i}$ denotes the coefficient-wise contribution of client $C_{i}$ to the joint secret polynomial. For notational clarity, the polynomial coefficient index is omitted; the sharing operations described below are applied independently to the secret-polynomial coefficients. The complete joint secret *s* is not explicitly constructed or stored during the protocol.

The public key pk is the final joined CKKS public key produced after completion of the *n*-party OpenFHE key-generation stage. This public key is associated with the implicit joint secret *s* in Equation (1) and is made available to the participating clients and the aggregation server. Each client encrypts its local model update under this common public key. For an encoded plaintext polynomial $\tilde{m}$, encryption produces

(2) $$c={\mathrm{Enc}}_{\mathrm{pk}}\left(\tilde{m}\right)=\left(c_{0},c_{1}\right).$$

For such a CKKS ciphertext, the corresponding decryption relation can be expressed approximately as

(3) $$c_{0}+c_{1}s\approx \tilde{m},$$

where the approximation accounts for the CKKS encoding and encryption noise. Equation (3) defines the quantity that the custom collaborative-decryption procedure must recover without explicitly constructing the complete joint secret *s*.

#### 4.1.2. Post-DKG Threshold-Share Construction

After the *n*-party key-establishment stage, each client $C_{i}$ reshapes its own local DKG secret contribution $s_{i}$ into client-specific subshares. For each coefficient of $s_{i}$, client $C_{i}$ constructs a polynomial of degree t−1,

(4) $$f_{i}\left(x\right)=s_{i}+\sum_{k=1}^{t-1}a_{i,k}x^{k},$$

where $f_{i}\left(0\right)=s_{i}$ and $a_{i,k}$ denotes a randomly generated sharing coefficient. Client $C_{i}$ evaluates this polynomial at the *n* distinct client indices:

(5) $$sh_{i,j}=f_{i}\left(j\right),\;j=1,\ldots ,n,$$

where $sh_{i,j}$ denotes the subshare generated by client $C_{i}$ for recipient client $C_{j}$.

An individual subshare $sh_{i,j}$ must be distinguished from the combined share ultimately associated with client $C_{j}$. After obtaining one subshare corresponding to each of the *n* DKG secret contributions, client $C_{j}$ forms

(6) $$S_{j}=\sum_{i=1}^{n}sh_{i,j}.$$

To show the relationship between $S_{j}$ and the implicit joint secret *s*, define

(7) $$F\left(x\right)=\sum_{i=1}^{n}f_{i}\left(x\right).$$

Because each $f_{i}\left(x\right)$ has degree at most t−1, the aggregate polynomial F(x) also has degree at most t−1. Furthermore,

(8) $$F\left(0\right)=\sum_{i=1}^{n}f_{i}\left(0\right)=\sum_{i=1}^{n}s_{i}=s,$$

while, for client $C_{j}$,

(9) $$F\left(j\right)=\sum_{i=1}^{n}f_{i}\left(j\right)=\sum_{i=1}^{n}sh_{i,j}=S_{j}.$$

Thus, $S_{j}=F\left(j\right)$ is the combined threshold share associated with client $C_{j}$ and with the implicit joint secret s=F(0). The combined share $S_{j}$ is not used to reconstruct and store *s*. Instead, client $C_{j}$ uses $S_{j}$ directly when computing its custom partial-decryption contribution, as described in Section 4.4. In the implementation, the coefficient representation of $S_{j}$ is embedded in an OpenFHE private-key-compatible polynomial container, enabling its use in custom polynomial arithmetic. This container represents the combined share $S_{j}$. Equations (1)–(9) define the implicit joint secret, public-key encryption and CKKS decryption relation, the coefficient-wise Shamir-based resharing procedure [41], the generation and combination of client subshares, and the relationship between the combined client shares and the implicit joint secret.

**Figure 2:**
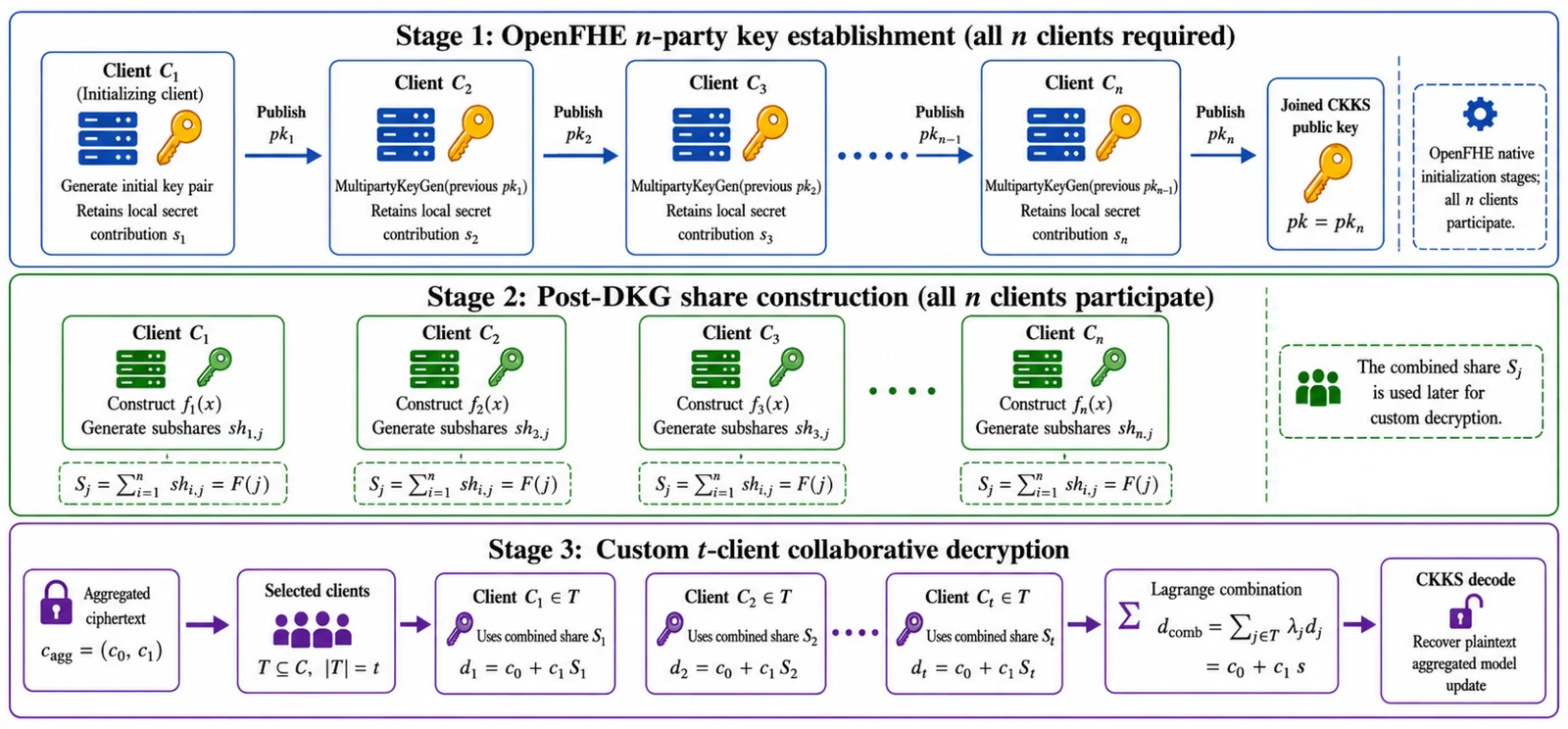
Cryptographic workflow of the proposed implementation showing (i) n-party OpenFHE key establishment, (ii) post-DKG construction of combined client shares, and (iii) custom t-client collaborative decryption.

The experimental implementation performs the OpenFHE key establishment and post-DKG share construction stages with all *n* registered clients. After these initialization stages have completed, a subset of *t* clients is selected for the custom collaborative-decryption procedure. Hence, the *t*-client property applies to the decryption stage after successful *n*-party initialization; it is not a claim that OpenFHE’s native multiparty DKG itself operates with only *t* of the *n* registered clients. Figure 2 summarizes these operations by distinguishing: (i) the *n*-party OpenFHE multiparty key-establishment stage that produces the joint public key pk; (ii) the post-DKG construction of the combined client shares $S_{j}$; and (iii) the custom *t*-client collaborative-decryption stage described in Section 4.4.

### 4.2. Threshold-Secured Federated Training and Encrypted Aggregation

After establishing the cryptographic infrastructure, the framework initiates privacy-preserving federated training using three benchmark datasets distributed across participating federated clients. Each federated client $C_{i}$ maintains a local dataset partition $D_{i}$ containing network-flow features associated with both benign and malicious traffic. The datasets include traffic statistics, protocol information, communication patterns, temporal flow characteristics, and packet-level behavioral features commonly used for intrusion detection. At federated round *r*, each client independently performs local optimization using the current global model parameters $w^{\left(r\right)}$. Following local training, the updated client-side model parameters are represented as

$$w_{i}^{\left(r+1\right)}.$$

To preserve confidentiality, the updated parameters are encrypted using the jointly generated public key:

(10) $$c_{i}={\mathrm{Enc}}_{pk}\left(w_{i}^{\left(r+1\right)}\right)$$

The encrypted model updates are subsequently transmitted to the aggregation server. Since the server operates under an honest-but-curious adversarial assumption, aggregation is performed exclusively within the encrypted domain without accessing plaintext model parameters. Using the additive homomorphic properties of the CKKS cryptosystem, the aggregation server computes:

(11) $$c_{agg}=\sum_{i=1}^{n}\alpha_{i}\cdot c_{i}={\mathrm{Enc}}_{pk}\left(\sum_{i=1}^{n}\alpha_{i}\cdot w_{i}^{\left(r+1\right)}\right),$$

where $\alpha_{i}$ denotes the aggregation weight associated with client $C_{i}$ subject to

(12) $$\sum_{i=1}^{n}\alpha_{i}=1.$$

The Equations (10)–(12) describe the encryption of local model updates, ciphertext-domain weighted aggregation, and normalization of the client aggregation weights. The aggregation server recovers only the aggregated global model required for the next federated learning round. At no stage does the server obtain the plaintext model updates of individual clients or possess sufficient cryptographic material to independently decrypt client-specific ciphertexts.

### 4.3. GRU-Based IDS Model Formulation

The proposed framework employs the same GRU-based intrusion detection model used in our previous HE-enabled federated IDS framework [13]. Maintaining an identical learning architecture enables a fair evaluation of the proposed OpenFHE-based threshold cryptographic framework by isolating the effects of decentralized key management and threshold decryption from changes in the underlying IDS model. The GRU architecture is retained for its computational efficiency and ability to capture temporal dependencies in SDN flow-based traffic. Moreover, compared with standard recurrent neural networks, GRUs use gating mechanisms to regulate information flow and mitigate vanishing-gradient effects, making them well-suited to learning sequential dependencies from SDN flow-based telemetry. For an input feature vector $x_{t}$ at time step *t*, the update gate is computed as

$$z_{t}=\sigma \left(W_{z}x_{t}+U_{z}h_{t-1}+b_{z}\right),$$

while the reset gate is defined as

$$r_{t}=\sigma \left(W_{r}x_{t}+U_{r}h_{t-1}+b_{r}\right).$$

The candidate hidden-state representation is then computed as

$${\tilde{h}}_{t}=\tanh\;\left(W_{h}x_{t}+U_{h}\left(r_{t}⊙h_{t-1}\right)+b_{h}\right),$$

where $W_{z}$, $W_{r}$, and $W_{h}$ denote trainable input weight matrices, $U_{z}$, $U_{r}$, and $U_{h}$ denote recurrent weight matrices, $b_{z}$, $b_{r}$, and $b_{h}$ are bias vectors, $h_{t-1}$ is the previous hidden state, σ(·) is the sigmoid activation function, and ⊙ denotes element-wise multiplication. The final hidden state is updated as

$$h_{t}=z_{t}⊙h_{t-1}+\left(1-z_{t}\right)⊙{\tilde{h}}_{t}.$$

Finally, the learned hidden representation is passed to a fully connected output layer for benign/attack classification. For binary classification, the output layer can be implemented using either a sigmoid neuron or a two-neuron softmax layer. The detailed architectural configuration of the GRU-based IDS model, including hidden-layer dimensions, batch size, optimizer settings, and federated training parameters, is summarized in in Section 5.

### 4.4. Threshold Collaborative Decryption

After encrypted aggregation, the aggregation server holds the aggregated CKKS ciphertext

$$c_{\mathrm{agg}}=\left(c_{0},c_{1}\right),$$

which encrypts the weighted aggregate of the participating clients’ local model updates under the joint public key pk defined in Section 4.1. The complete joint secret *s* is not explicitly reconstructed for decryption. Instead, the combined client shares $S_{j}$ constructed in Section 4.1 are used directly in the custom collaborative-decryption procedure. Let

T⊆C,|T|=t,

denote the set of clients selected for decryption. In the reported experiments, the threshold follows the strict-majority setting

$$t=\left\lfloor \frac{n}{2}\right\rfloor +1,$$

yielding the evaluated configurations (3,4), (5,8), and (7,12). These values are design choices of the proposed experimental implementation rather than threshold parameters provided by OpenFHE’s native CKKS multiparty decryption. Each selected client $C_{j}\in T$ holds its combined share $S_{j}=F\left(j\right)$. Rather than invoking OpenFHE’s native functions for the reported *t*-client decryption path, the custom C++/Pybind implementation computes the partial-decryption polynomial

(13) $$d_{j}=c_{0}+c_{1}S_{j}.$$

Thus, $d_{j}$ is generated directly from the aggregated ciphertext and client $C_{j}$’s combined share $S_{j}$. For the participating set T, the Lagrange coefficient associated with client $C_{j}$ is [42]

(14) $$\lambda_{j}=\prod_{\begin{array}{c} \ell \in T\\ \ell \neq j \end{array}}\frac{-\ell }{j-\ell }.$$

Since $S_{j}=F\left(j\right)$ and F(x) has degree at most t−1, interpolation over the *t* selected client indices gives

(15) $$\sum_{j\in T}\lambda_{j}S_{j}=F\left(0\right)=s.$$

The same Lagrange coefficients satisfy [42]

(16) $$\sum_{j\in T}\lambda_{j}=1.$$

The aggregation server therefore combines the received partial-decryption polynomials as

(17) $$\begin{array}{cc} d_{\mathrm{comb}} & =\sum_{j\in T}\lambda_{j}d_{j}\\ & =\sum_{j\in T}\lambda_{j}\left(c_{0}+c_{1}S_{j}\right)\\ & =c_{0}\sum_{j\in T}\lambda_{j}+c_{1}\sum_{j\in T}\lambda_{j}S_{j}\\ & =c_{0}+c_{1}s. \end{array}$$

By comparison with Equation (3), $d_{\mathrm{comb}}$ is the CKKS plaintext-bearing polynomial associated with the joint secret *s*. It can therefore be passed to the CKKS decoding procedure to recover the approximate plaintext aggregated model update. Importantly, Lagrange interpolation is applied directly to the partial-decryption values; the complete joint secret *s* is not explicitly reconstructed or stored during this operation. In the implementation, the combined polynomial is placed in the plaintext-bearing ciphertext component and the second component is set to zero before invoking the OpenFHE decoding path. Thus, the secret-dependent operation has already been performed through the custom partial-decryption and Lagrange-combination steps. Because F(x) has degree at most t−1, *t* distinct evaluations are sufficient to determine F(0) through the interpolation procedure used in the implementation. The aggregation server itself holds no combined client share $S_{j}$ and therefore cannot independently execute the custom decryption using only its locally held material. It nevertheless remains the protocol coordinator that collects the authorized partial-decryption contributions and performs their combination. After decoding, the recovered global model $w^{\left(r+1\right)}$ is distributed to the participating federated clients to initialize the next communication round. The same encrypted training and collaborative decryption procedure is repeated until the federated learning process converges. Protection against arbitrary-ciphertext, repeated-decryption, or decryption-oracle requests from a malicious coordinator is not provided by the present implementation and is addressed separately in Section 3.3.4.

### 4.5. Real-Time SDN Deployment and Online Inference

After federated training and offline explainability analysis, the optimized global GRU-based IDS model is deployed for real-time SDN traffic monitoring and online intrusion detection. During runtime, live traffic is monitored and converted into flow-level feature representations, which are then preprocessed and passed to the trained global IDS model for benign/attack classification. The deployed IDS operates as an external monitoring and inference module integrated with the SDN environment. The Ryu controller manages OpenFlow forwarding, while Zeek-generated traffic records are transferred to the preprocessing and GRU inference pipeline for real-time classification.

Importantly, computationally expensive cryptographic operations, including distributed key generation, encrypted aggregation, and collaborative threshold decryption, are confined to the offline phase of federated training. Similarly, SHAP-based explainability analysis is conducted offline to avoid additional online inference delay. Therefore, the real-time deployment phase uses the converged plaintext global model for low-latency inference. This separation between offline secure federated training and online IDS inference enables practical deployment in SDN environments while preserving runtime responsiveness.

## 5. Experimental Setup

This section presents the experimental environment, federated learning configuration, CKKS cryptographic settings, SDN deployment architecture, and evaluation methodology used to validate the proposed threshold-secured federated IDS framework. The experimental design is developed to comprehensively evaluate not only detection capability, but also cryptographic robustness, explainability, scalability, and real-time operational feasibility under realistic SDN deployment conditions.

### 5.1. Experiment Environment Setup

The framework is implemented using a hybrid Python–C++ architecture integrating federated deep learning, threshold-secured HE, explainability analysis, and real-time SDN traffic monitoring. The experiments are conducted on a high-performance workstation equipped with GPU acceleration to support federated model training and cryptographic computation. Table 3 summarizes the hardware and software environment used throughout the experiments.

The FL pipeline, IDS training, and SHAP-based explainability analysis are implemented in Python using TensorFlow and Keras. Threshold HE, distributed key generation, encrypted aggregation, and collaborative decryption are implemented using OpenFHE with the CKKS encryption scheme. The custom C++ extension additionally implements Shamir-based secret-share construction and Lagrange-based t-out-of-n partial-decryption reconstruction over the OpenFHE CKKS ciphertext representation. Pybind11 is used to establish interoperability between the Python-based federated learning pipeline and the C++-based cryptographic implementation [43].

### 5.2. Distributed Federated Learning Configuration

The IDS framework uses a GRU-based federated learning architecture to capture the temporal and sequential characteristics of SDN traffic flows. The experiments are conducted under multiple distributed client configurations to evaluate scalability and collaborative learning behavior. Table 4 summarizes the federated learning configuration used throughout the experiments.

The experiments are conducted using three benchmark intrusion-detection datasets, namely InSDN [44], CICDDoS2017 [45], and CICDDoS2019 [46]. To ensure a fair comparison with our previous federated learning framework, the same preprocessed datasets are employed throughout this work. The preprocessing pipeline—including data cleaning, missing-value handling, duplicate removal, categorical feature encoding, feature normalization, class balancing, and feature selection—follows the methodology presented in our previous studies [13,47]. Consequently, the improvements reported in this paper are attributable to the proposed threshold-secured cryptographic framework rather than changes in data preprocessing or feature engineering.

These datasets contain diverse benign and malicious traffic behaviors, enabling comprehensive evaluation under heterogeneous network conditions. The datasets are distributed across multiple federated clients in configurations with 4, 8, and 12 clients. These configurations enabled comprehensive analysis of collaborative learning behavior, cryptographic overhead growth, communication cost, and threshold-decryption scalability as the federated network size increased. The selected GRU architecture effectively models the sequential and temporal traffic characteristics commonly observed in SDN environments. Batch normalization and dropout layers are incorporated to improve training stability, accelerate convergence, and mitigate overfitting during federated optimization.

### 5.3. Threshold Homomorphic Encryption Configuration

The proposed framework utilizes the CKKS approximate homomorphic encryption scheme implemented using OpenFHE. OpenFHE is an open-source homomorphic encryption library that supports advanced lattice-based schemes, including CKKS, BFV, and BGV, as well as multiparty and threshold functionalities. OpenFHE provides the multiparty CKKS and distributed key generation primitives used in this work. The arbitrary *t*-out-of-*n* reconstruction threshold is enforced through the custom C++/pybind11 layer implementing Shamir-based secret-share construction and Lagrange-based combination, as described in Section 4.1. These capabilities enable the implementation of a decentralized trust architecture in which no single entity possesses the complete secret key, thereby eliminating the centralized trusted-dealer dependency commonly found in conventional homomorphic-encryption-enabled federated learning frameworks. Table 5 summarizes the cryptographic parameters used throughout the experiments.

The selected cryptographic parameters are carefully configured to balance ciphertext precision, computational efficiency, and secure aggregation capability. The chosen ring dimension and scaling parameters provide sufficient numerical precision for encrypted federated aggregation while maintaining feasible runtime performance in real-time SDN deployments. Unlike centralized homomorphic encryption architectures, the framework distributes cryptographic trust among participating clients through DKG. Each participant maintains an independent secret-key share, while the global secret key is never reconstructed in plaintext form. Collaborative decryption, therefore, requires participation from at least *t* clients, ensuring resistance against single-party compromise and honest-but-curious aggregation servers.

### 5.4. SDN Deployment Environment

To validate the framework’s practical deployment feasibility, a real-time SDN testbed is implemented using Mininet, the Ryu SDN controller, and the Zeek network monitoring framework. The testbed is designed to emulate live SDN traffic conditions and evaluate the runtime behavior of the proposed IDS under both benign and adversarial scenarios. Although the framework is evaluated on the InSDN, CICDDoS2017, and CICDDoS2019 datasets during offline federated training and detection performance analysis, the real-time SDN deployment experiment uses the converged global model trained on the InSDN dataset. This choice is motivated by the SDN-specific nature of InSDN, which better reflects SDN traffic characteristics and attack behaviors relevant to controller-based deployment.

**Figure 3:**
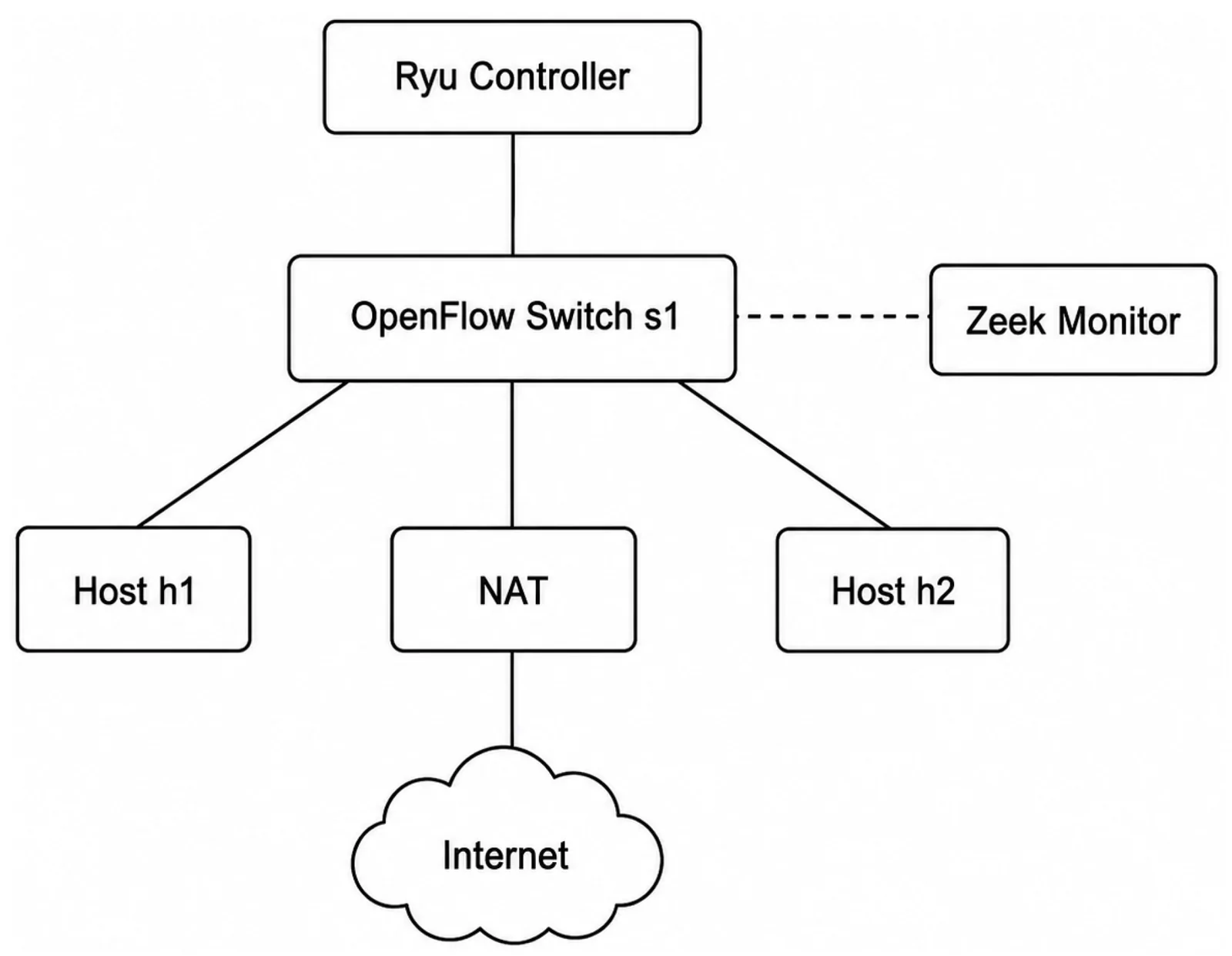
Mininet-based SDN testbed topology used for real-time deployment evaluation.

**Figure 4:**
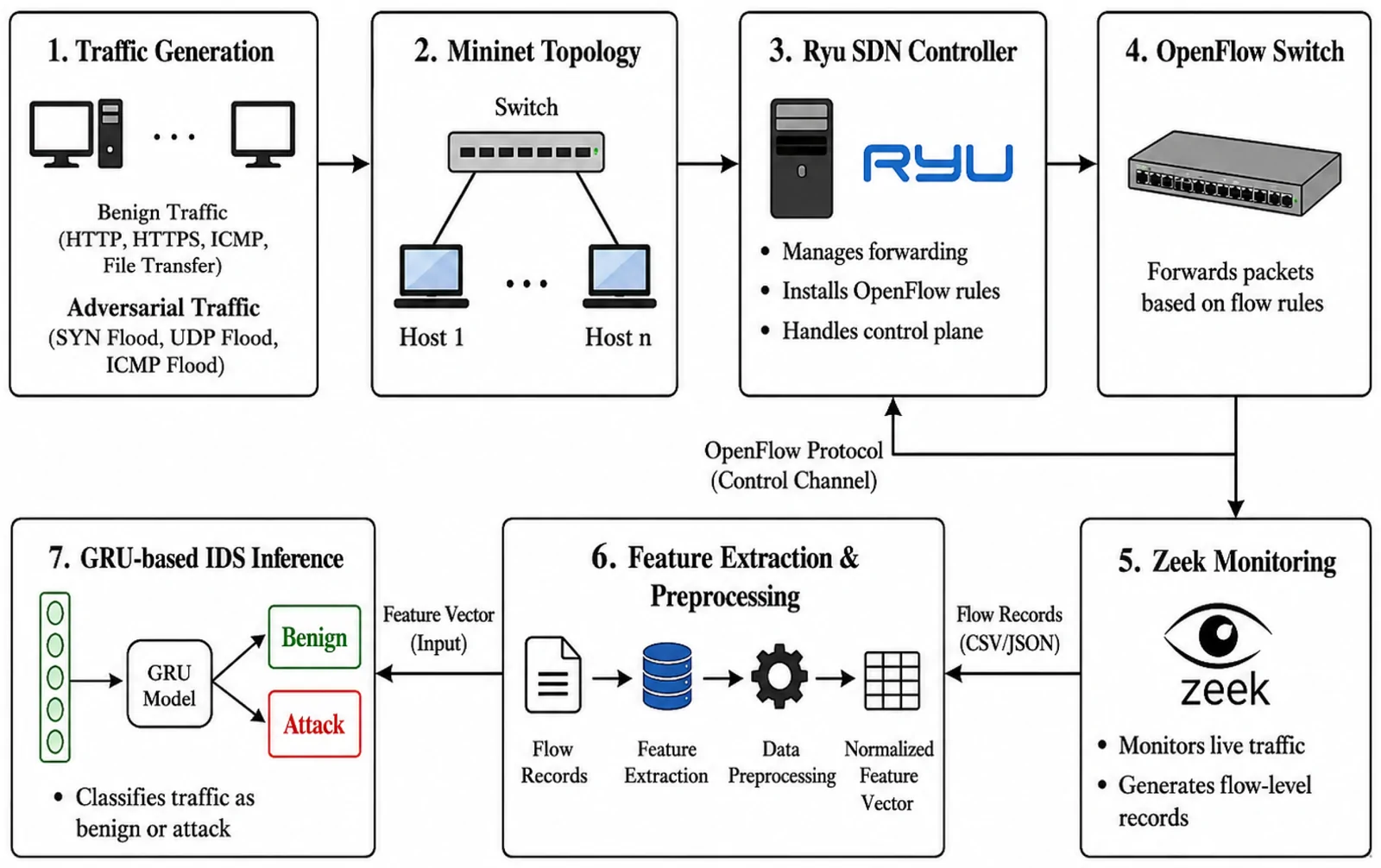
Real-time SDN integration workflow showing traffic generation, Mininet topology, Ryu-based OpenFlow control, Zeek traffic monitoring, feature extraction and preprocessing, and GRU-based benign/attack classification.

The Mininet testbed is configured using a lightweight single-switch topology. As illustrated in Figure 3, the topology consists of two hosts connected to a single OpenFlow-enabled virtual switch with NAT-based Internet connectivity [48]. The Ryu controller manages the forwarding behavior of the OpenFlow switch, while Zeek monitors runtime traffic and extracts flow-level telemetry for intrusion-detection analysis. The operational workflow of the real-time IDS deployment is shown in Figure 4. The workflow includes traffic generation, Mininet-based SDN emulation, OpenFlow switching and Ryu controller interaction, Zeek-based traffic monitoring, flow-level feature extraction, preprocessing, GRU-based inference using the trained global model, and final benign/attack prediction.

During deployment evaluation, two traffic scenarios are generated to emulate realistic SDN operating conditions. In the benign scenario, normal traffic is generated through repeated HTTP/HTTPS requests, ICMP exchanges, and file transfers to represent standard user activity. In the adversarial scenario, high-volume SYN, UDP, and ICMP flood attacks are launched to evaluate the robustness of the IDS pipeline under volumetric attack conditions. These traffic streams are processed through the Mininet-based topology, monitored by Zeek, transformed into flow-level features, preprocessed, and then classified in real time by the trained global GRU model. Overall, the runtime deployment environment is designed to evaluate the operational feasibility of the proposed framework under live SDN conditions, including detection performance, inference latency, throughput, CPU utilization, runtime stability, and behavior under both benign and adversarial traffic scenarios.

### 5.5. SDN Controller and IDS Integration

The Ryu controller is connected to the OpenFlow-enabled Mininet switch and manages forwarding via the OpenFlow protocol. Traffic generated by the Mininet hosts flows through the virtual switch, while Zeek continuously monitors it and produces flow-level records. The Python-based IDS module collects these records, converts them into the feature format required by the trained GRU model, and processes them through the deployment preprocessing pipeline. The resulting feature vectors are then passed to the converged global GRU model for benign or malicious classification. Thus, the Ryu controller manages the SDN forwarding plane, while Zeek and the external IDS module handle traffic monitoring, feature extraction, preprocessing, and model inference. In the current prototype, the prediction results are used to evaluate real-time intrusion detection and are not automatically translated into OpenFlow mitigation rules.

### 5.6. Evaluation Metrics

To comprehensively evaluate the threshold-secured federated intrusion-detection framework, the experimental analysis considers four major dimensions: detection effectiveness, cryptographic performance, explainability, and real-time deployment efficiency.

#### 5.6.1. Detection Performance Metrics

The detection performance of the presented IDS framework is evaluated using standard classification metrics, including accuracy, precision, recall, F1-score, Matthews correlation coefficient (MCC), Cohen’s Kappa coefficient, logarithmic loss, ROC analysis, AUC, and confusion matrix analysis. Let TP, TN, FP, and FN denote true positives, true negatives, false positives, and false negatives, respectively. Accuracy measures the overall classification correctness of the IDS model and is computed as

(18) $$\mathrm{Accuracy}=\frac{TP+TN}{TP+TN+FP+FN}.$$

Precision evaluates the proportion of correctly predicted attack samples among all samples predicted as attacks:

(19) $$\mathrm{Precision}=\frac{TP}{TP+FP}.$$

Recall measures the capability of the IDS to correctly identify malicious traffic flows:

(20) $$\mathrm{Recall}=\frac{TP}{TP+FN}.$$

The F1-score represents the harmonic mean of precision and recall:

(21) $$F1\text{-}\mathrm{Score}=2\times \frac{\mathrm{Precision}\times \mathrm{Recall}}{\mathrm{Precision}+\mathrm{Recall}}.$$

To provide a balanced evaluation under potentially imbalanced traffic distributions, the Matthews Correlation Coefficient (MCC) is additionally employed:

(22) $$\mathrm{MCC}=\frac{TP\times TN-FP\times FN}{\sqrt{\left(TP+FP)(TP+FN)(TN+FP)(TN+FN\right)}}.$$

Furthermore, Cohen’s Kappa coefficient is used to evaluate the agreement between predicted and actual classifications beyond random chance:

(23) $$\kappa =\frac{p_{o}-p_{e}}{1-p_{e}},$$

where (p_o) and (p_e) denote the observed and expected agreement probabilities, respectively. Equations (18)–(23) define the accuracy, precision, recall, F1-score, Matthews correlation coefficient, and Cohen’s Kappa metrics used in the evaluation. In addition, logarithmic loss is analyzed to assess the confidence of probabilistic predictions and the stability of model convergence. ROC and AUC analyses are conducted to evaluate the discriminative capability of the proposed IDS across varying classification thresholds, while confusion matrix analysis is used to examine class-wise prediction behavior and misclassification characteristics.

#### 5.6.2. Cryptographic Performance Metrics

To evaluate the computational and communication overhead introduced by threshold-secured homomorphic encryption and distributed key generation, cryptographic performance analysis is conducted using encryption latency, encrypted aggregation overhead, collaborative threshold-decryption latency, and communication overhead. The analysis further investigates scalability behavior under varying federated client distributions and compares the proposed OpenFHE-based framework against the conventional TenSEAL-based architecture presented in our previous work [13].

#### 5.6.3. Explainability Evaluation Metrics

To evaluate interpretability and operational transparency, a SHAP-based explainability analysis is conducted using global feature importance, local explanations of model decisions, and class-wise interpretability. These evaluations analyze how traffic features contribute to IDS prediction behavior across diverse SDN attack scenarios.

#### 5.6.4. Real-Time Deployment Performance Metrics

The framework’s runtime feasibility is evaluated in a real-time SDN environment using Mininet, the Ryu controller, and Zeek-based traffic monitoring. The deployment analysis considered inference latency, end-to-end pipeline latency, CDF (Cumulative Distribution Function) latency behavior, throughput-versus-latency characteristics, CPU utilization trends, and runtime stability evaluation. Overall, these evaluation metrics enable a comprehensive assessment of detection effectiveness, cryptographic robustness, explainability, scalability, and the feasibility of practical deployment within privacy-preserving SDN intrusion-detection environments.

The goal of the deployment experiments is to verify the operational feasibility of the proposed online inference pipeline after threshold-secured federated training, rather than to benchmark extremely large-scale multi-domain SDN infrastructures. Large-scale federated deployments involving substantially larger client populations remain an important direction for future investigation.

## 6. Results Analysis

This section presents a comprehensive evaluation of the threshold-secured federated intrusion detection framework. The experiments are conducted using multiple federated configurations to assess the framework’s scalability and robustness under varying client distributions.

### 6.1. Detection Performance Analysis

**Figure 5:**
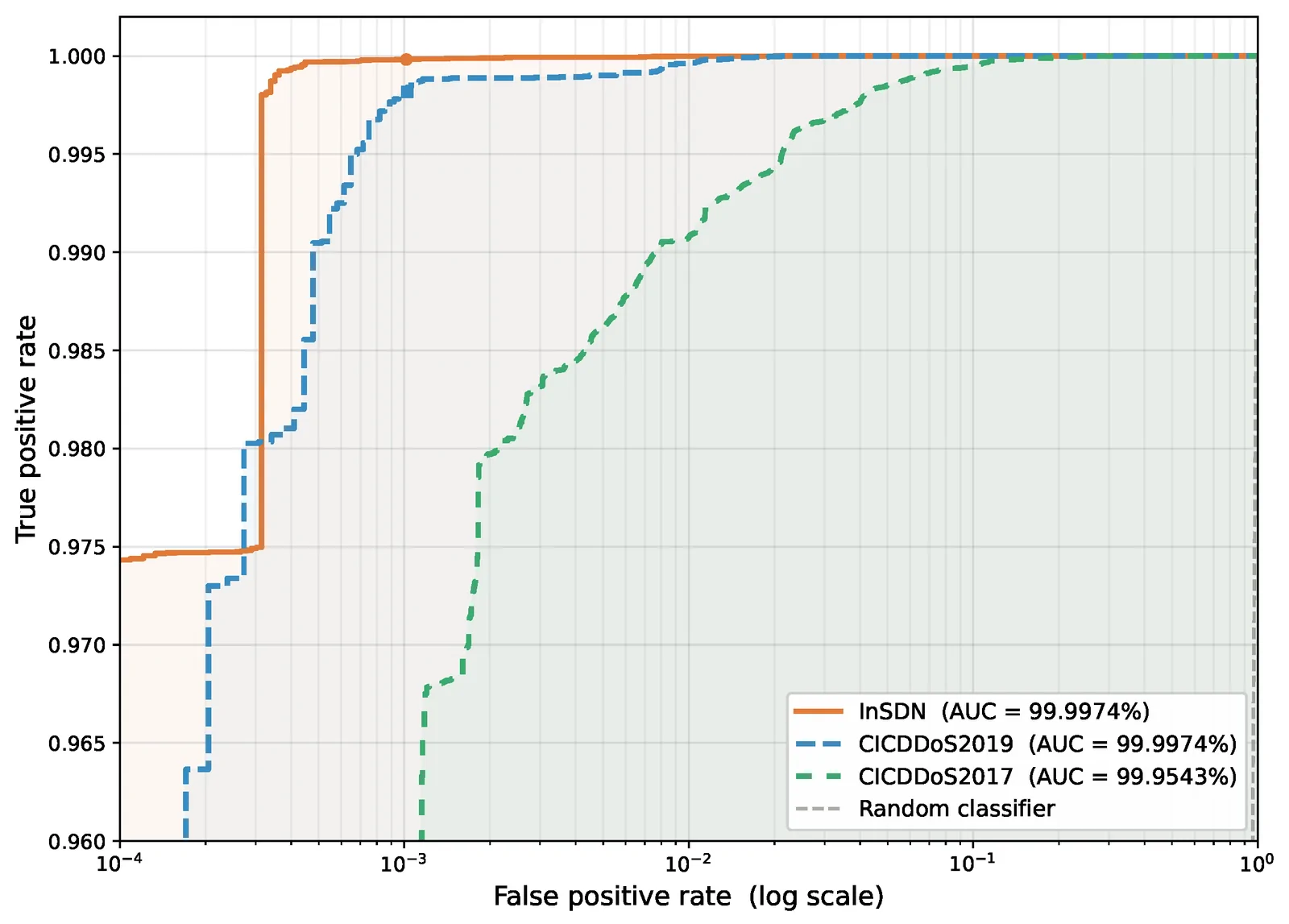
ROC curves of the proposed IDS model evaluated on the InSDN, CICDDoS2019, and CICDDoS2017 datasets using a logarithmic false-positive-rate scale.

**Figure 6:**
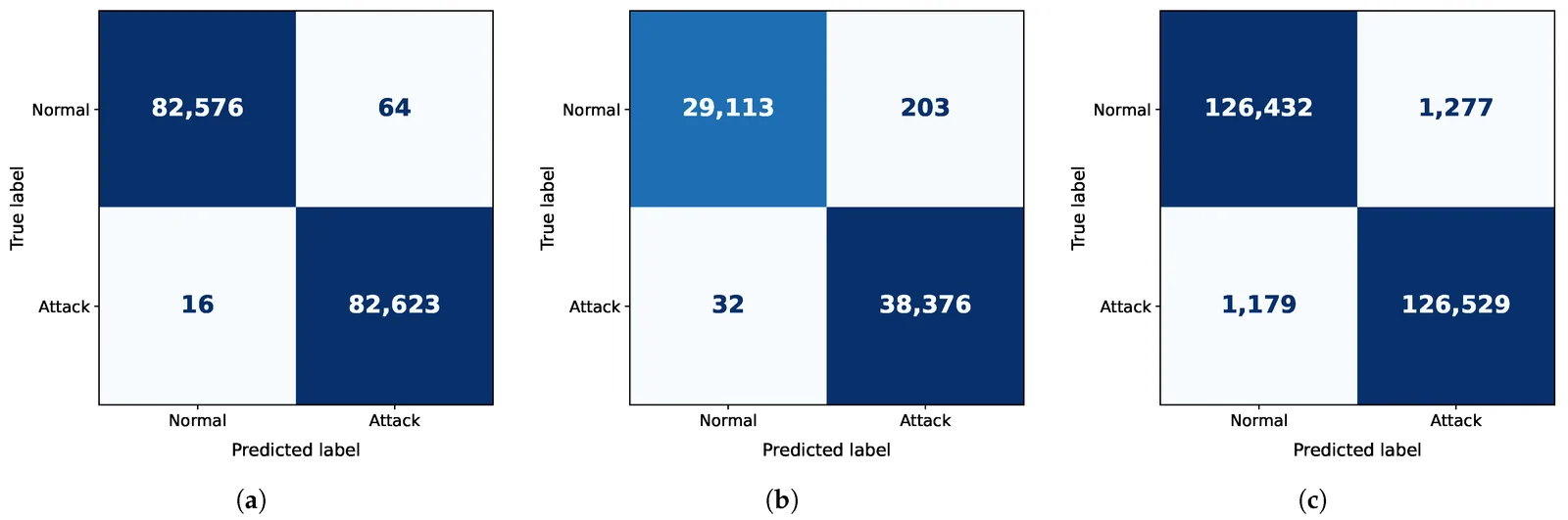
Confusion matrices of the proposed IDS model for (**a**) InSDN, (**b**) CICDDoS2019, and (**c**) CICDDoS2017. The results show high classification performance with few false-positive and false-negative predictions across the evaluated SDN intrusion-detection datasets.

The performance of the threshold-secured federated IDS framework on the InSDN, CICDDoS2017, and CICDDoS2019 datasets. Table 6 summarizes overall detection performance, while Figure 5 and Figure 6 illustrate the ROC characteristics and confusion matrices, respectively.

#### 6.1.1. Quantitative Detection Performance

The quantitative detection performance of the IDS framework on three datasets is shown in Table 6. The results demonstrate consistently high intrusion-detection performance across all evaluated datasets. Specifically, the proposed IDS achieved accuracies of 99.95%, 99.03%, and 99.65% for InSDN, CICDDoS2017, and CICDDoS2019, respectively. Since accuracy is computed from both correctly detected benign and attack samples, these results indicate that the majority of SDN traffic flows are correctly classified across heterogeneous datasets. However, accuracy alone may be insufficient for IDS evaluation, particularly when benign and attack classes are imbalanced. Therefore, precision, recall, F1-score, MCC, and Cohen’s Kappa are jointly analyzed to assess the reliability of the classification.

Moreover, precision values above 99% indicate that only a very small fraction of flows predicted as attacks are actually benign, confirming a low false-positive rate. This is important for SDN environments because excessive false alarms can trigger unnecessary flow-rule updates and increase controller overhead. Similarly, recall values above 99% confirm that the model successfully detects most malicious flows, minimizing false negatives that could allow attacks to go undetected. The consistently high F1-scores demonstrate that the framework maintains a strong balance between reducing false positives and maintaining attack-detection sensitivity.

Among the evaluated datasets, InSDN achieved the strongest overall performance, with 99.95% accuracy, 99.92% precision, 99.98% recall, and 99.95% F1-score. These values are consistent with its confusion matrix, in which the number of misclassified samples is extremely small relative to the total number of evaluated flows. The CICDDoS2019 dataset also achieved strong performance, particularly in recall, indicating that the model can detect large-scale DDoS attack traffic with very few missed attack samples. Although CICDDoS2017 produced slightly lower performance, the model still maintained 99.03% accuracy and 99.03% F1-score, demonstrating robustness under more diverse traffic and attack variations.

The MCC and Cohen’s Kappa values further verify the reliability of the IDS framework. MCC evaluates the correlation between predicted and actual classes by jointly considering TP, TN, FP, and FN, while Cohen’s Kappa measures agreement beyond random chance. The near-unity MCC and Kappa values show that the proposed framework maintains balanced classification performance rather than relying on majority-class dominance. In addition, the low log-loss values indicate that the model produces confident and well-calibrated probabilistic predictions. Overall, the mathematical and empirical results confirm that the threshold-secured federated learning process preserves high intrusion-detection capability while providing decentralized cryptographic protection.

#### 6.1.2. ROC Analysis

Figure 5 presents the ROC curves of our IDS framework across three evaluated datasets. The ROC curves remain concentrated near the upper-left region, indicating high true-positive rates under extremely low false-positive conditions. The framework achieved AUC scores of 99.9974% on both the InSDN and CICDDoS2019 datasets, and 99.9543% on the CICDDoS2017 dataset. Notably, the ROC curves are presented on a logarithmic scale of false-positive rate to improve visualization in extremely small false-positive regions. This representation is particularly important for SDN IDS systems because excessive false alarms can undermine controller stability and dynamic flow-rule management.

Additionally, the InSDN dataset exhibited the steepest ROC transition, indicating rapid convergence toward optimal detection performance with minimal false alarms. Similarly, the CICDDoS2019 dataset demonstrated near-identical behavior, confirming strong generalization capability across large-scale DDoS traffic patterns. Although the CICDDoS2017 ROC curve increased more gradually in the extremely low false-positive region, it still converged near the ideal operating point, demonstrating robust detection capability under highly heterogeneous traffic conditions.

#### 6.1.3. Confusion Matrix Analysis

The confusion matrices shown in Figure 6 further validate the reliability of the framework under heterogeneous SDN traffic conditions. For the InSDN dataset shown in Figure 6a, the model correctly classified 82,576 benign flows and 82,623 attack flows, while generating only 64 false positives and 16 false negatives. These results demonstrate an extremely reliable intrusion discrimination capability with minimal classification errors.

Similarly, for the CICDDoS2019 dataset shown in Figure 6b, the model correctly identified 29,113 benign flows and 38,376 attack flows while producing only 203 false positives and 32 false negatives, confirming stable classification behavior across large-scale attack traffic scenarios. For the CICDDoS2017 dataset shown in Figure 6c, the model correctly detected 126,432 benign samples and 126,529 attack samples. Although the dataset yielded comparatively higher false-positive and false-negative counts, the overall detection performance remained highly stable, given the significantly larger sample size and broader diversity of attacks in CICDDoS2017. Overall, the experimental results confirm that the proposed federated IDS achieves highly reliable intrusion-detection performance across multiple SDN security datasets while maintaining stable classification behavior under heterogeneous traffic distributions. The combination of high recall and extremely low false-positive rates further demonstrates the framework’s suitability for practical, real-time SDN security applications.

### 6.2. Cryptographic Performance Analysis

This section evaluates the computational and communication overhead of the threshold-secured FL-based IDS framework. The analysis compares the OpenFHE-based threshold cryptographic architecture with the conventional TenSEAL-based homomorphic encryption framework presented in our previous work [13]. The evaluation is conducted on the InSDN, CICDDoS2017, and CICDDoS2019 datasets across multiple federated client configurations with 4, 8, and 12 distributed participants. The analysis considers encryption latency, encrypted aggregation overhead, threshold collaborative decryption latency, and communication overhead to investigate scalability behavior under decentralized trust settings. To mathematically interpret the cryptographic overhead, let *n* denote the number of federated clients, $S_{w}$ denote the size of each local model update, α denote the ciphertext expansion factor introduced by homomorphic encryption, and *t* denote the minimum number of clients required for threshold decryption. The total cryptographic latency of one secure federated aggregation round is

$$T_{\mathrm{crypto}}=T_{\mathrm{enc}}+T_{\mathrm{agg}}+T_{\mathrm{pdec}}+T_{\mathrm{comb}},$$

where $T_{\mathrm{enc}}$, $T_{\mathrm{agg}}$, $T_{\mathrm{pdec}}$, and $T_{\mathrm{comb}}$ represent encryption, encrypted aggregation, partial decryption, and final share-combination latency, respectively. Since each client encrypts its local update independently, the total encryption cost scales as

$$T_{\mathrm{enc}}^{\mathrm{total}}=\sum_{i=1}^{n}T_{\mathrm{enc}}^{\left(i\right)}.$$

Similarly, the threshold decryption cost depends on the number of participating clients and can be written as

$$T_{\mathrm{dec}}^{\mathrm{threshold}}=\sum_{j=1}^{t}T_{\mathrm{pdec}}^{\left(j\right)}+T_{\mathrm{comb}}.$$

These relationships explain why cryptographic overhead increases as the federated network grows from 4 to 8 and 12 clients.

#### 6.2.1. Encryption Overhead Analysis

**Figure 7:**
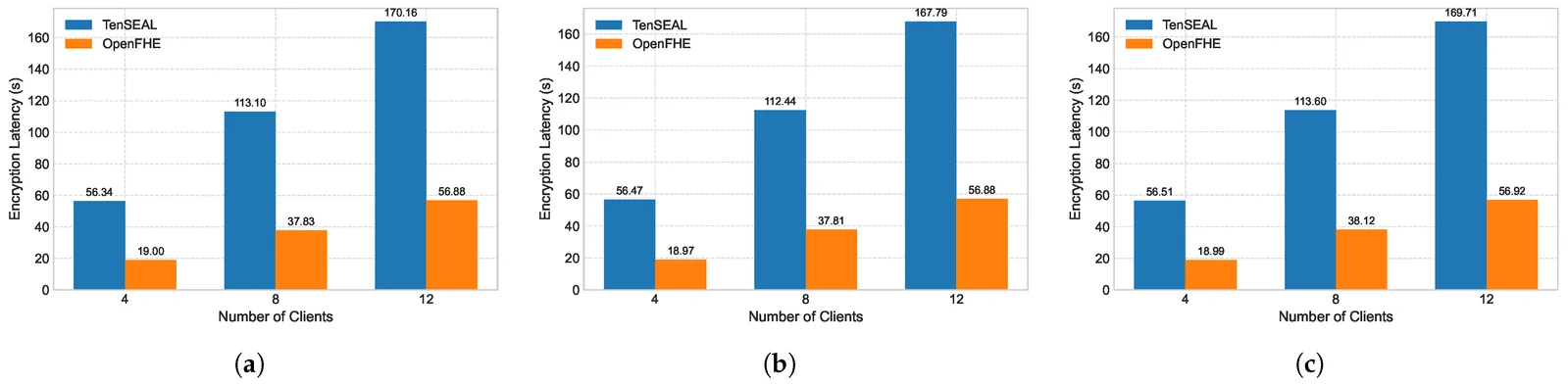
Encryption latency comparison between TenSEAL and OpenFHE under varying numbers of federated clients. OpenFHE demonstrates lower encryption latency across all evaluated client configurations. (**a**) InSDN; (**b**) CICDDoS2019; (**c**) CICDDoS2017.

The encryption latency comparison between the proposed OpenFHE-based framework and the conventional TenSEAL-based architecture across different federated client configurations is shown in Figure 7. The experimental results demonstrate that the OpenFHE-based framework consistently achieves substantially lower encryption latency than TenSEAL across all evaluated datasets and client scales. For the InSDN dataset, OpenFHE required 19.00 s, 37.83 s, and 56.88 s for 4-, 8-, and 12-client configurations, respectively, whereas TenSEAL required 56.34 s, 112.10 s, and 170.16 s under identical conditions. Similar performance trends are observed for CICDDoS2017 and CICDDoS2019.

The results indicate that encryption overhead scales approximately linearly with the number of participating federated clients due to the increasing volume of encrypted model parameters generated during local training. This behavior can be mathematically interpreted as

$$T_{\mathrm{enc}}^{\mathrm{total}}\left(n\right)\approx n\cdot T_{\mathrm{enc}}^{\mathrm{avg}},$$

where $T_{\mathrm{enc}}^{\mathrm{avg}}$ is the average encryption time required for one client to encrypt its local model update. Therefore, increasing the client population from 4 to 8 and 12 increases the total encryption workload approximately in proportion to *n*, which aligns with the latency trend shown in Figure 7.

Nevertheless, the OpenFHE framework maintains significantly lower encryption latency because of its optimized polynomial-ring arithmetic, efficient CKKS encoding implementation, and its optimized native C++ CKKS implementation. Across the evaluated client configurations, OpenFHE reduced encryption latency by approximately threefold compared with the TenSEAL-based implementation. This improvement demonstrates that decentralized threshold cryptographic operations can be achieved without introducing prohibitive client-side encryption latency, thereby improving the feasibility of secure federated IDS operation under the evaluated client configurations.

#### 6.2.2. Encrypted Aggregation Overhead

**Figure 8:**
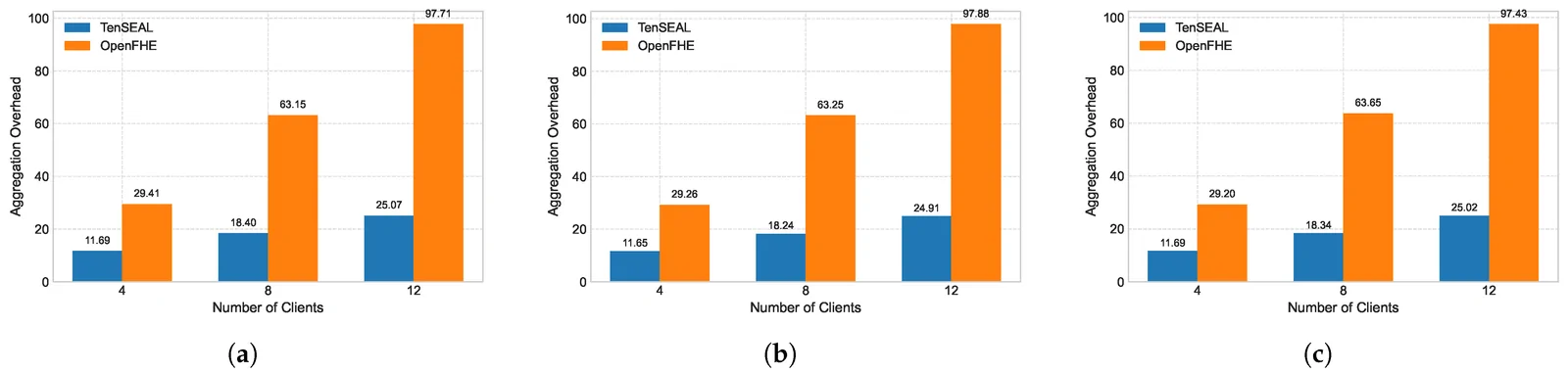
Aggregation overhead comparison between TenSEAL and OpenFHE under varying numbers of federated clients. (**a**) InSDN; (**b**) CICDDoS2019; (**c**) CICDDoS2017.

Figure 8 illustrates the encrypted aggregation overhead measured at the central aggregation server during federated model update integration. Unlike encryption latency, the OpenFHE-based framework incurs higher aggregation overhead compared with the TenSEAL baseline. For example, under the InSDN dataset with 12 federated clients, OpenFHE required 97.71 s for encrypted aggregation, whereas TenSEAL required only 25.07 s. Similar scalability behavior is observed across CICDDoS2017 and CICDDoS2019. For *n* encrypted client updates, the encrypted aggregation operation can be written as

$$\mathrm{Enc}\left(W^{r+1}\right)=\sum_{i=1}^{n}\frac{m_{i}}{M}\;\mathrm{Enc}\left(W_{i}^{r}\right),$$

where $W_{i}^{r}$ is the local model update of client *i* at round *r*, $m_{i}$ is the number of local samples, and $M=\sum_{i=1}^{n}m_{i}$. Since the server aggregates directly in the ciphertext domain, the aggregation cost increases with both the number and size of encrypted updates. Thus, the server-side overhead can be approximated as

$$T_{\mathrm{agg}}\left(n\right)\propto n\cdot C_{\mathrm{HE}}\left(S_{w}\right),$$

where $C_{\mathrm{HE}}\left(S_{w}\right)$ denotes the homomorphic computation cost for an encrypted update of size $S_{w}$. The increased aggregation overhead is expected, as the proposed framework performs homomorphic operations within a threshold-secure cryptographic environment. In contrast to conventional single-key HE architectures, threshold-secured aggregation requires additional ciphertext transformations, multiparty evaluation-key management, and collaborative cryptographic operations for distributed key generation and threshold evaluation.

Furthermore, aggregation overhead increases progressively as the number of federated participants grows from 4 to 12 clients. This behavior reflects the increased complexity of ciphertext processing and the expanded collaborative cryptographic interaction required in larger decentralized federated environments. Although OpenFHE introduces higher aggregation latency than TenSEAL, the additional overhead reflects the operational cost of removing centralized complete-secret-key custody and independent single-party decryption. Consequently, the aggregation overhead remains an acceptable trade-off for achieving distributed key custody under the stated trust assumptions within privacy-preserving SDN intrusion-detection systems.

#### 6.2.3. Threshold Collaborative Decryption Latency

**Figure 9:**
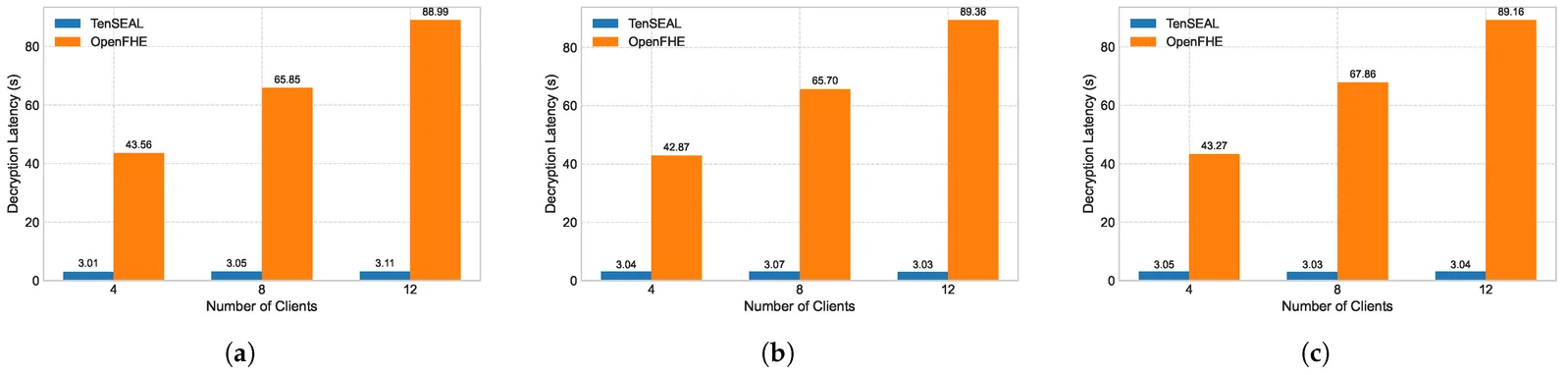
Decryption latency comparison between TenSEAL and OpenFHE under varying numbers of federated clients. (**a**) InSDN; (**b**) CICDDoS2019; (**c**) CICDDoS2017.

Figure 9 compares the decryption latency of the OpenFHE-based threshold cryptographic framework against the conventional TenSEAL architecture. The experimental results reveal the most substantial performance divergence during the decryption phase. TenSEAL maintains nearly constant decryption latency across all client configurations, ranging from approximately 3 s to 3.11 s. However, this low overhead is achieved because TenSEAL relies on a centralized secret-key architecture in which a single trusted authority performs direct decryption of ciphertext.

In contrast, the proposed OpenFHE framework employs threshold collaborative decryption, which increases decryption latency as the federated network grows. For the InSDN dataset, OpenFHE required 43.56 s, 65.85 s, and 88.99 s for 4-, 8-, and 12-client configurations, respectively. A similar trend is observed for CICDDoS2017 and CICDDoS2019. This increased overhead arises because threshold decryption requires multiple participating clients to independently generate partial decryption shares, which are then combined through collaborative reconstruction. Therefore, the aggregation server cannot decrypt encrypted model parameters using its own key material and requires partial decryption contributions from at least *t* participating clients.

In the threshold setting, the aggregation server cannot decrypt the aggregated ciphertext $C_{\mathrm{agg}}$ using a single secret key. Instead, each participating client *j* generates a partial decryption share $d_{j}$, and at least *t* valid shares are required to reconstruct the plaintext aggregated update:

$$W^{r+1}=\mathrm{Combine}\;\left(d_{1},d_{2},\ldots ,d_{t}\right).$$

The security condition can be expressed as

$$|C|<t\;\Rightarrow \;\mathrm{Pr}[\mathrm{Dec}\left(C_{\mathrm{agg}}\right)]\approx 0,$$

where |C| denotes the number of colluding or compromised participants. This means that fewer than *t* clients cannot recover the aggregated plaintext update. Therefore, the increased decryption latency observed in Figure 9 reflects the computational cost of replacing centralized decryption with threshold-secured collaborative decryption. Although collaborative decryption incurs higher runtime overhead than centralized decryption, it removes the single point of complete secret-key custody present in conventional single-key HE-FL systems. The resulting increase in latency, therefore, represents a deliberate and necessary trade-off to preserve decentralized trust and resist honest-but-curious aggregation servers.

#### 6.2.4. Communication Overhead Analysis

**Figure 10:**
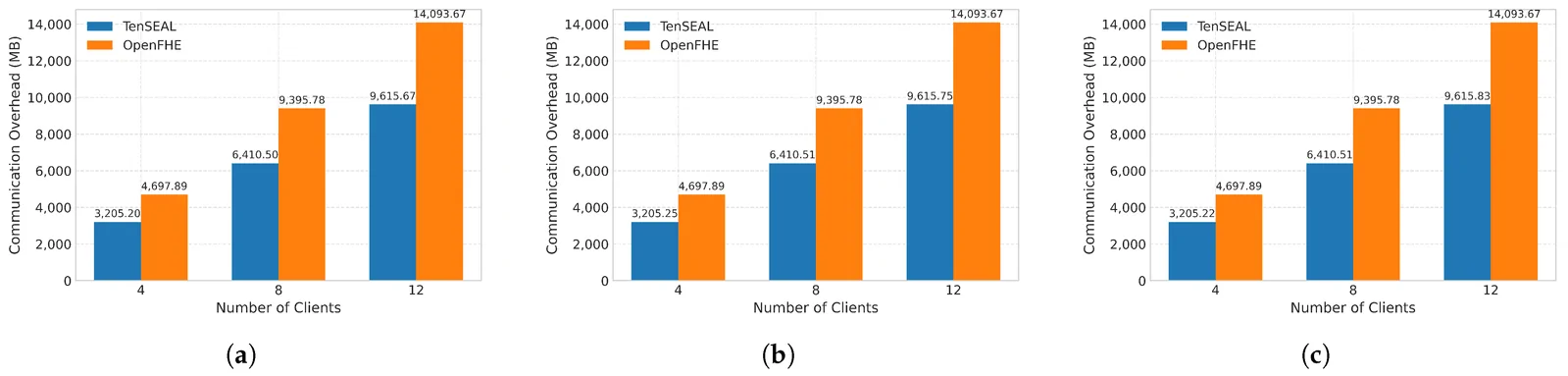
Communication Overhead comparison between TenSEAL and OpenFHE under varying numbers of federated clients. (**a**) InSDN; (**b**) CICDDoS2019; (**c**) CICDDoS2017.

The communication overhead comparison between OpenFHE and TenSEAL across varying federated client configurations is shown in Figure 10. The results indicate that the OpenFHE framework incurs higher communication overhead than the TenSEAL baseline across all datasets. For example, under the 12-client configuration, OpenFHE required approximately 14,093 MB of communication overhead, whereas TenSEAL required approximately 9616 MB.

The increased communication cost primarily results from the distributed nature of threshold cryptographic operations. In addition to encrypted model updates, the OpenFHE-based framework requires transmitting distributed public-key shares, evaluation keys, partial decryption shares, and collaborative reconstruction messages among participating clients and the aggregation server. The communication overhead can be approximated as

$$C_{\mathrm{total}}=n\alpha S_{w}+C_{\mathrm{key}}+tS_{d}+C_{\mathrm{coord}},$$

where $n\alpha S_{w}$ represents the transmission cost of encrypted model updates from *n* clients, $C_{\mathrm{key}}$ denotes the communication required for distributed key generation and evaluation-key material, $tS_{d}$ represents the transmission cost of *t* partial decryption shares, and $C_{\mathrm{coord}}$ denotes the additional coordination messages required for threshold cryptographic operations. In contrast, a conventional single-key HE–FL system primarily requires encrypted update transmission and centralized decryption, resulting in lower communication overhead but weaker distribution of cryptographic trust.

Moreover, communication overhead increases proportionally with the number of federated clients because larger collaborative environments require additional cryptographic coordination and secure multiparty interactions. Despite this increase, the communication overhead remains operationally feasible in practical federated SDN environments, particularly because cryptographic synchronization occurs during the offline federated training stage rather than during real-time packet forwarding.

#### 6.2.5. Security–Performance Trade-Off Discussion

The comparative analysis demonstrates a clear security–performance trade-off between the previous TenSEAL-based architecture and the proposed OpenFHE-based threshold framework. The latter introduces higher encrypted-aggregation, collaborative-decryption, and communication overhead because it replaces centralized secret-key ownership with dealerless distributed key generation and *t*-out-of-*n* collaborative decryption. These additional costs arise from both the threshold protocol and its implementation-level cryptographic processing.

In the TenSEAL-based architecture, decryption is performed using a single secret key:

$$W^{r+1}={\mathrm{Dec}}_{sk}\left(C_{\mathrm{agg}}\right),$$

where $C_{\mathrm{agg}}$ denotes the aggregated ciphertext and sk is the centralized secret key. Although this approach reduces decryption latency, compromising the secret-key holder may expose the aggregated federated model update. In contrast, the proposed framework requires at least *t* valid partial decryption contributions. Neither the aggregation server nor an individual client can independently decrypt the aggregated update using its locally held key material.

For a controlled comparison, both implementations used the same GRU model, model-update dimensions, polynomial modulus degree of 8192, CKKS packing size of 4096, effective multiplicative depth, scaling precision, client configuration, communication rounds, and hardware environment. Therefore, the opposite latency trends are not caused by different cryptographic parameters.

The lower OpenFHE encryption latency is attributable to packed CKKS encoding and public-key encryption performed through optimized native C++ operations exposed by the pybind11 extension. During aggregation, however, serialized ciphertext chunks are transferred from Python to C++, deserialized, multiplied by the client weight, added to the running ciphertext, and serialized again. Repeating these operations for every chunk and participating client increases aggregation latency. By comparison, TenSEAL performs encrypted-vector operations through a higher-level in-memory tensor representation. The higher OpenFHE communication overhead primarily results from its larger serialized ciphertext representation.

As summarized in Table 7, OpenFHE reduces encryption latency from 170.16 s to 56.88 s, while aggregation, collaborative decryption, and communication costs increase. The principal benefit of the proposed framework is therefore not uniformly lower latency, but the removal of centralized secret-key custody and support for *t*-out-of-*n* collaborative decryption while maintaining detection accuracy above 99% across the three evaluated datasets.

### 6.3. Explainability Analysis

Although deep learning-based IDS systems can achieve high classification performance, their decision-making processes often remain opaque in operational SDN environments. To improve transparency and build analyst-level trust, the OpenFHE-based federated IDS framework integrates SHAP-based explainability. SHAP quantifies each traffic feature’s contribution to the final prediction using cooperative game theory. For a given input sample (x), the model output can be expressed as

$$f\left(x\right)=\phi_{0}+\sum_{i=1}^{M}\phi_{i},$$

where f(x) denotes the model prediction, $\phi_{0}$ is the baseline prediction, $\phi_{i}$ is the contribution of the *i*-th feature, and *M* is the total number of traffic features. For global interpretation, the importance of feature *j* is computed as the mean absolute SHAP value across *N* samples:

$$I_{j}=\frac{1}{N}\sum_{k=1}^{N}\left|\phi_{j}^{\left(k\right)}\right|.$$

Thus, features with larger $I_{j}$ values contribute more strongly to the overall IDS decision behavior. This formulation enables both global and local interpretation of intrusion-detection decisions and helps identify the SDN traffic characteristics that drive benign and malicious classifications.

Beyond model interpretation, the SHAP module is intended to support operational decision-making in SDN security management. Global SHAP explanations identify recurring traffic characteristics that influence the IDS across multiple samples, helping analysts prioritize monitoring policies and validate whether the model relies on security-relevant features. Local explanations provide instance-specific evidence for individual alerts, allowing analysts to examine the dominant features behind a benign or malicious prediction before applying actions such as traffic blocking, flow-rule modification, or further forensic investigation. In the present framework, SHAP is used as a decision-support mechanism rather than as an automatic mitigation trigger.

#### 6.3.1. Global and Ranked Feature Importance Analysis

**Figure 11:**
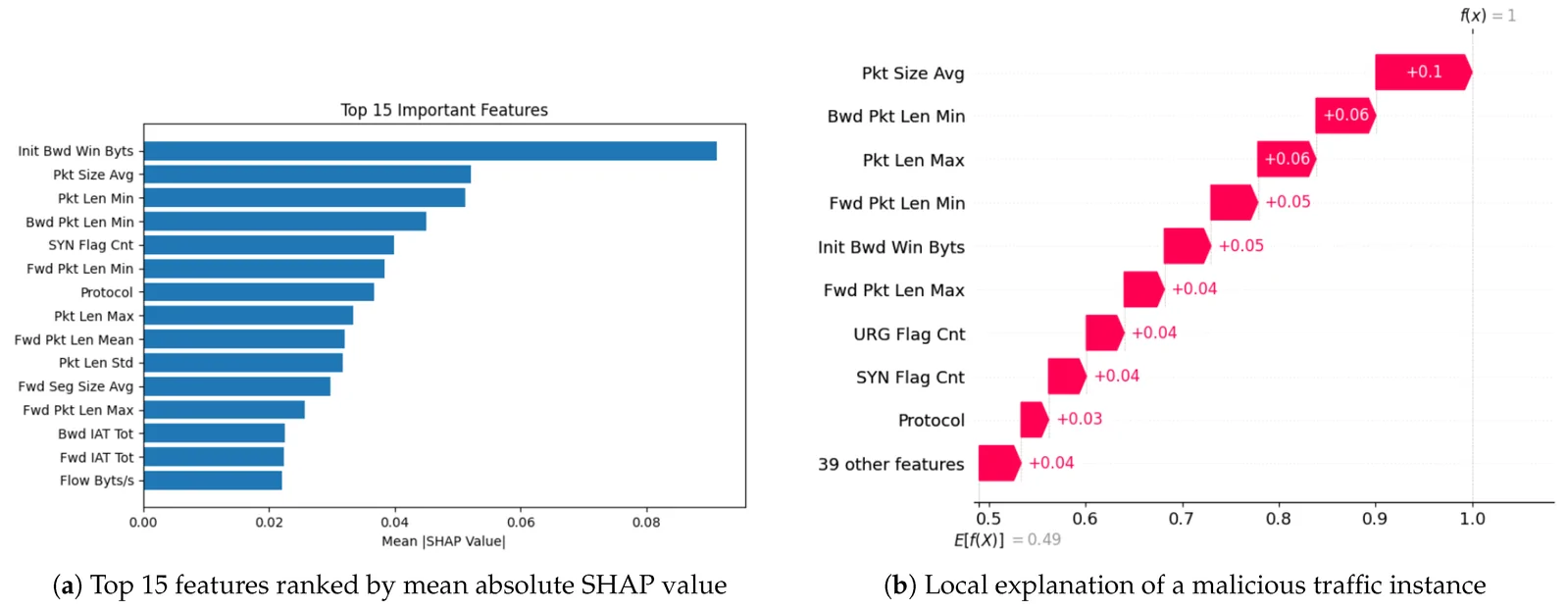
SHAP-based global and local explainability analysis of the proposed federated IDS framework: (**a**) the 15 most influential features ranked by their mean absolute SHAP values; (**b**) a local SHAP waterfall explanation for a malicious SDN traffic instance.

To analyze the overall decision behavior of the presented IDS framework, global SHAP analysis is conducted on the evaluation dataset. Figure 11a presents the top-ranked traffic features by mean absolute SHAP value, and Table 8 provides the corresponding security interpretation.

The results indicate that packet-level statistics, transport-layer signaling behavior, and temporal flow characteristics are the dominant feature groups learned by the federated IDS model. Among all evaluated features, *Init_Bwd_Win_Byts* has the highest mean SHAP magnitude, indicating that backward flow-control behavior provides strong discriminative information for distinguishing benign and malicious SDN communication patterns. Packet-size-related features, including *Pkt_Size_Avg*, *Pkt_Len_Min*, and *Bwd_Pkt_Len_Min*, also show high SHAP contributions, suggesting that irregular packet-size distributions are important indicators of flooding attacks, burst-oriented traffic generation, and anomalous communication behavior.

In addition, protocol-level indicators such as *SYN_Flag_Cnt* contribute strongly to the model output, confirming that abnormal TCP handshake behavior remains highly relevant for detecting malicious SDN traffic. Temporal flow metrics, including *Flow_Byts/s*, *Flow_Pkts/s*, and inter-arrival-time-related features, further indicate that the model captures burstiness and abnormal communication dynamics commonly observed in DDoS attacks. The gradual reduction in SHAP magnitude across lower-ranked features indicates that the model relies primarily on a compact subset of highly informative traffic attributes rather than on noisy or redundant indicators.

Overall, the consistency between SHAP-derived feature rankings and known SDN attack characteristics indicates that our framework learns semantically meaningful intrusion patterns rather than relying on arbitrary statistical correlations.

#### 6.3.2. Local Explanation of Model Decisions

While global explanations provide insight into overall model behavior, local explanations enable interpretation of individual traffic predictions. To investigate instance-level IDS decisions, SHAP waterfall analysis is performed on representative samples of malicious traffic. The results are shown in Figure 11b.

The waterfall analysis shows that the selected traffic instance is classified as malicious with high confidence, as the cumulative SHAP contributions shift the model output from the baseline value E[f(X)]=0.49 toward the attack class, where f(x)≈1. The baseline prediction initially starts near

E[f(X)]=0.49,

and progressively shifts toward the attack class through the cumulative effect of multiple abnormal traffic features. Among the dominant contributors, *Pkt_Size_Avg* has the largest positive influence on the final prediction, increasing the attack confidence score by approximately +0.10. Similarly, *Bwd_Pkt_Len_Min* and *Pkt_Len_Max* each contribute approximately +0.06, while *Fwd_Pkt_Len_Min* and *Init_Bwd_Win_Byts* further increase the malicious prediction score by nearly +0.05.

Additionally, protocol-oriented indicators, including *SYN_Flag_Cnt* and *URG_Flag_Cnt*, positively contribute to the final attack prediction, underscoring the importance of abnormal TCP signaling behavior in identifying malicious SDN communication. The cumulative contribution of these features indicates that the IDS framework makes logically consistent, security-relevant decisions based on meaningful traffic characteristics rather than arbitrary statistical artifacts. This instance-level interpretability is particularly valuable for forensic investigation and real-time SDN security monitoring.

#### 6.3.3. Fidelity-Based Explanation Validation

**Figure 12:**
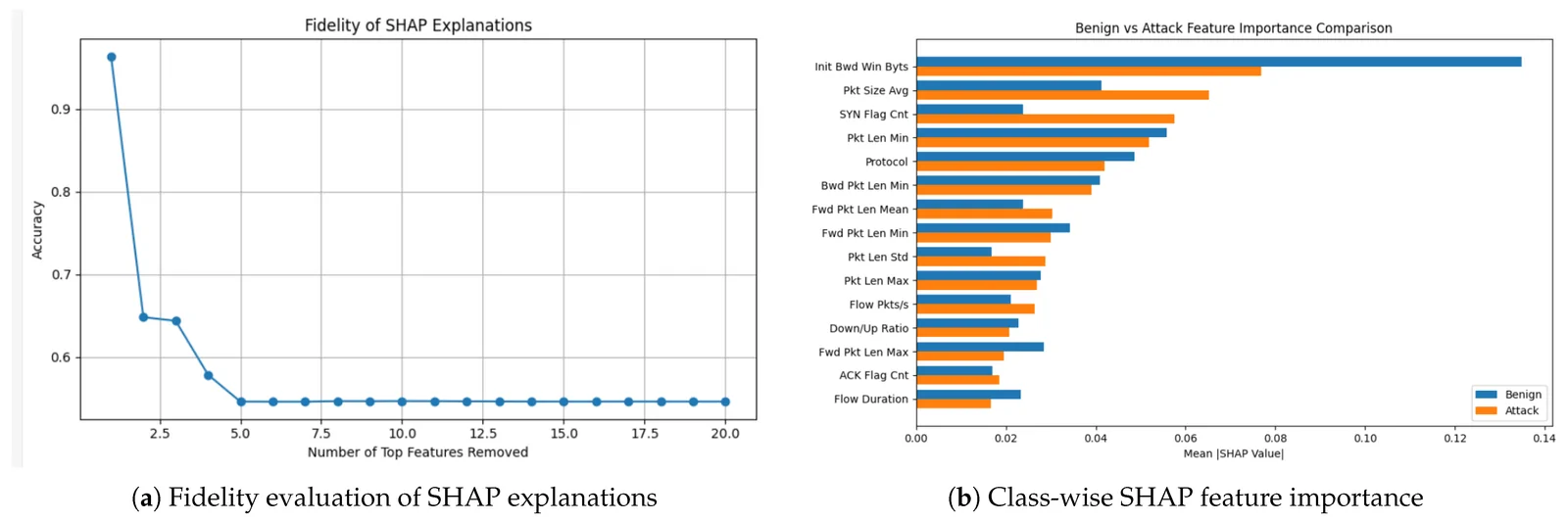
SHAP fidelity and attack-wise explainability analysis: (**a**) accuracy degradation following the progressive masking the top SHAP-ranked features; (**b**) class-wise feature-importance analysis performed on malicious SDN traffic samples.

To evaluate the reliability of the SHAP explanations, a fidelity analysis is conducted by progressively masking the top SHAP-ranked features and measuring the resulting degradation in classification performance, as shown in Figure 12a. The SHAP feature ranking used in this analysis is obtained from the converged 4-client global model trained on the InSDN dataset. InSDN is selected because it represents the SDN-specific operating environment targeted by the proposed framework. SHAP values are computed using a subset of 200 held-out InSDN test samples and a background set of 100 training samples. These SHAP values are then used to rank the most influential features.

The fidelity evaluation itself is performed on the complete held-out InSDN test set. Table 6 reports an accuracy of 99.95% on this full test set when all features are retained. In Figure 12a, the first reported fidelity point corresponds to masking the single highest-ranked SHAP feature, after which the accuracy decreases to approximately 95%. After masking the two highest-ranked features, the accuracy further drops to nearly 65%. As additional dominant features are masked, classification performance continues to deteriorate, reaching approximately 54–55% after masking the top five ranked features. This degradation can be interpreted as a fidelity score that measures the dependence of the model output on the most influential SHAP-ranked features [49]:

$$\Delta Acc\left(k\right)=Acc_{\mathrm{all}}-Acc_{-Top(k)},$$

where $Acc_{\mathrm{all}}$ denotes the accuracy obtained using all features, and $Acc_{-Top(k)}$ denotes the accuracy after removing the top *k* SHAP-ranked features. A larger ΔAcc(k) indicates that the removed features are genuinely important for the model’s prediction behavior. Therefore, the sharp decline in accuracy after removing the top-ranked features confirms that the SHAP explanations faithfully reflect the model’s decision process. If the explanations are unreliable, removing the highest-ranked features would not produce such a substantial performance drop. These findings demonstrate that the IDS relies on a compact, meaningful set of discriminative SDN traffic features rather than on noisy or redundant attributes.

#### 6.3.4. Class-Wise Explanation Analysis

To further investigate class-specific decision behavior, SHAP analysis is performed separately for benign and malicious traffic samples, as illustrated in Figure 12b. The results reveal clear differences between benign and attack traffic across several important features. In particular, *Pkt_Size_Avg* shows a stronger contribution for malicious traffic, indicating that abnormal packet-size behavior is a key indicator of attack-oriented communication. Similarly, *SYN_Flag_Cnt* exhibits higher SHAP magnitude for attack samples, confirming that abnormal TCP signaling and incomplete handshake behavior are strongly associated with SDN-targeted attacks.

Interestingly, *Init_Bwd_Win_Byts* contributes more to benign traffic than to malicious traffic. This suggests that stable bidirectional flow-control behavior provides useful evidence of legitimate SDN communication, helping the model distinguish normal from anomalous traffic patterns. Additional flow-level indicators, including *Flow_Pkts/s*, *Flow_Duration*, and *Pkt_Len_Std*, further underscore the importance of burst-oriented communication and temporal irregularities during malicious activity.

Overall, the class-wise SHAP analysis confirms that the proposed threshold-secured federated IDS captures meaningful traffic signatures for both benign and attack classes. The alignment between SHAP-derived explanations and known SDN attack characteristics strengthens the framework’s interpretability, reliability, and practical suitability for real-time SDN security monitoring.

#### 6.3.5. Stability Across Federated Client Configurations

To evaluate whether the explanations remain stable as the federation size changes, SHAP analysis is repeated using the converged global models trained with 4, 8, and 12 clients. All three models are evaluated using the same held-out InSDN samples, identical preprocessing parameters, and the same SHAP background set. Global feature importance is calculated using the mean absolute SHAP value of each feature. Explanation stability is quantified using Spearman rank correlation across the complete feature-importance rankings, the number of common features among the ten highest-ranked features, and Jaccard similarity between the corresponding top-10 feature sets.

As shown in Table 9, the global SHAP explanations remained highly consistent across the three federated configurations. Spearman rank correlations ranged from 0.905 to 0.944, and all correlations are statistically significant at *p* < 0.001. The 4-client model shared eight of its ten most influential features with both the 8-client and 12-client models, corresponding to a Jaccard similarity of 0.667. The 8-client and 12-client models contained the same ten highest-ranked features, yielding a Jaccard similarity of 1.000, although their internal feature ordering differed slightly.

The feature-level results further showed that *Init Bwd Win Byts* remained the most influential feature across all three configurations. Other consistently important features included *Pkt Len Min*, *Pkt Len Max*, *Bwd Pkt Len Min*, *Pkt Size Avg*, and *SYN Flag Cnt*. These findings indicate that increasing the number of participating clients from 4 to 12 did not materially alter the IDS’s principal feature-level reasoning.

### 6.4. SDN Integration and Real-Time Deployment Evaluation

This section evaluates the SDN integration and runtime behavior of the proposed federated IDS framework under live network traffic. The deployed pipeline includes Mininet-based traffic generation, OpenFlow forwarding via the Ryu-controlled switch, Zeek-based traffic monitoring, flow-level feature extraction and preprocessing, and online inference with the converged global GRU model. Unlike offline dataset evaluation, this experiment assesses how the trained federated model performs in an SDN monitoring environment under practical latency and resource constraints. The evaluation therefore considers real-time detection effectiveness, inference latency, throughput scalability, CPU utilization, and runtime stability under both benign and adversarial traffic.

#### 6.4.1. Real-Time Detection Performance

**Figure 13:**
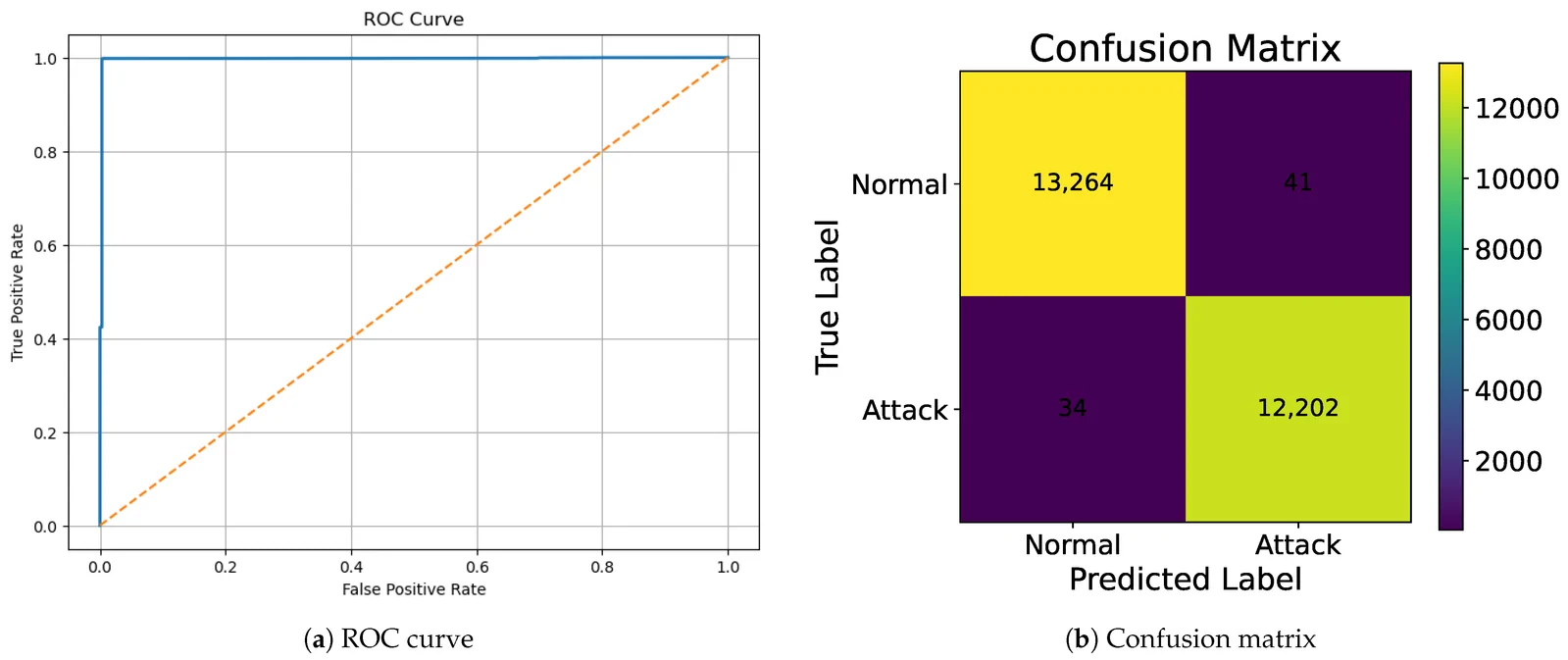
Real-time detection performance of the proposed threshold-secured federated IDS during SDN deployment: (**a**) receiver operating characteristic curve demonstrating strong class separability and high detection capability; (**b**) confusion matrix showing accurate classification of benign and malicious traffic flows with few false predictions.

The real-time deployment evaluation shows that our federated IDS framework maintains strong discriminative capability under operational SDN conditions. Figure 13a presents the ROC curve from the live deployment evaluation, while Figure 13b shows the corresponding confusion matrix. The ROC curve approaches the upper-left corner, indicating high true-positive performance with low false-positive rates. This result demonstrates that the deployed IDS preserves strong class separability between benign and malicious traffic even under continuous real-time traffic monitoring and online inference constraints.

The confusion matrix further confirms the effectiveness of deployment. Specifically, the model accurately classifies 13,264 benign flows and 12,202 malicious flows, with only 41 false positives and 34 false negatives. Based on these values, the real-time accuracy, precision, recall, and F1-score are 99.71%, 99.67%, 99.72%, and 99.69%, respectively, as summarized in Table 10. The false-positive rate (FPR) is only 0.31%, which is important for operational SDN environments because excessive false alarms can overwhelm network administrators and trigger unnecessary flow-rule updates. Similarly, the false-negative rate (FNR) is only 0.28%, indicating that the deployed IDS misses very few malicious flows even under adversarial traffic conditions.

Overall, the real-time detection analysis shows that the proposed offline, threshold-based federated training process does not compromise runtime detection effectiveness. After encrypted federated training and collaborative threshold decryption, only the final global GRU-based IDS model is deployed in the SDN environment for live inference. As a result, resource-intensive cryptographic operations are removed from the real-time detection process, enabling fast normal/attack classification in practical SDN conditions.

#### 6.4.2. Latency Distribution Analysis

**Figure 14:**
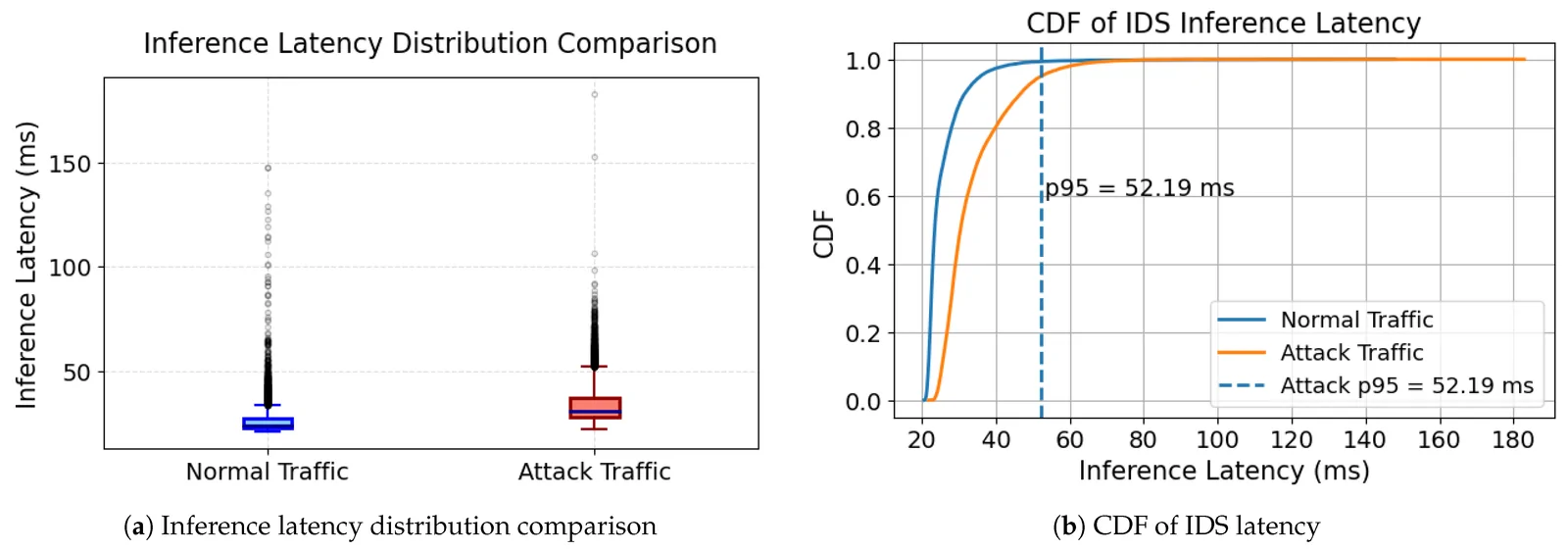
Runtime inference-latency analysis under normal and attack traffic conditions: (**a**) boxplot comparison of the inference-latency distributions, showing increased variance under attack traffic; (**b**) cumulative distribution function of IDS latency, demonstrating bounded runtime responsiveness with an attack-traffic 95th-percentile latency of approximately 52 ms.

To investigate runtime responsiveness, inference latency distributions are analyzed under both normal and attack traffic conditions using box plots and cumulative distribution functions, as illustrated in Figure 14a and Figure 14b, respectively.

The boxplot in Figure 14a shows the runtime inference latency distribution under normal and attack traffic conditions. It indicates that normal traffic yields a compact latency distribution with a lower median inference delay, reflecting stable runtime behavior during normal SDN operation. In contrast, attack traffic produces a higher median latency and a wider interquartile range, reflecting the additional processing burden from increased flow activity, Zeek-based parsing, feature extraction, and inference scheduling during adversarial traffic generation.

Although both normal and attack scenarios contain latency outliers, the majority of inference operations remain within a practical low-latency range. The attack-traffic distribution exhibits more extreme outliers, attributable to burst-oriented malicious traffic and temporary processing contention during high-volume SYN, UDP, and ICMP flood generation.

The CDF in Figure 14b further confirms the bounded runtime behavior of the model. Normal-traffic inference operations complete rapidly, with most predictions finishing within approximately 40ms. Under attack traffic, the latency distribution shifts moderately to the right; however, the 95th percentile latency remains 52.19ms. This indicates that 95% of attack-traffic inference operations are completed within approximately 52ms, demonstrating that the model preserves near real-time responsiveness even under adversarial traffic conditions. The online detection delay can be expressed as

$$T_{\mathrm{online}}=T_{\mathrm{feature}}+T_{\mathrm{infer}}+T_{\mathrm{decision}},$$

where $T_{\mathrm{feature}}$, $T_{\mathrm{infer}}$, and $T_{\mathrm{decision}}$ denote the feature-extraction, model-inference, and decision-generation latencies, respectively. Since DKG, encryption, encrypted aggregation, and threshold decryption are performed during offline federated training, they are excluded from $T_{\mathrm{online}}$. This separation explains why our framework can maintain bounded inference latency during real-time SDN deployment. Overall, the latency analysis confirms that the deployed GRU inference pipeline and live SDN traffic-monitoring process maintain bounded runtime responsiveness at the evaluated testbed scale.

#### 6.4.3. Throughput and Scalability Analysis

**Figure 15:**
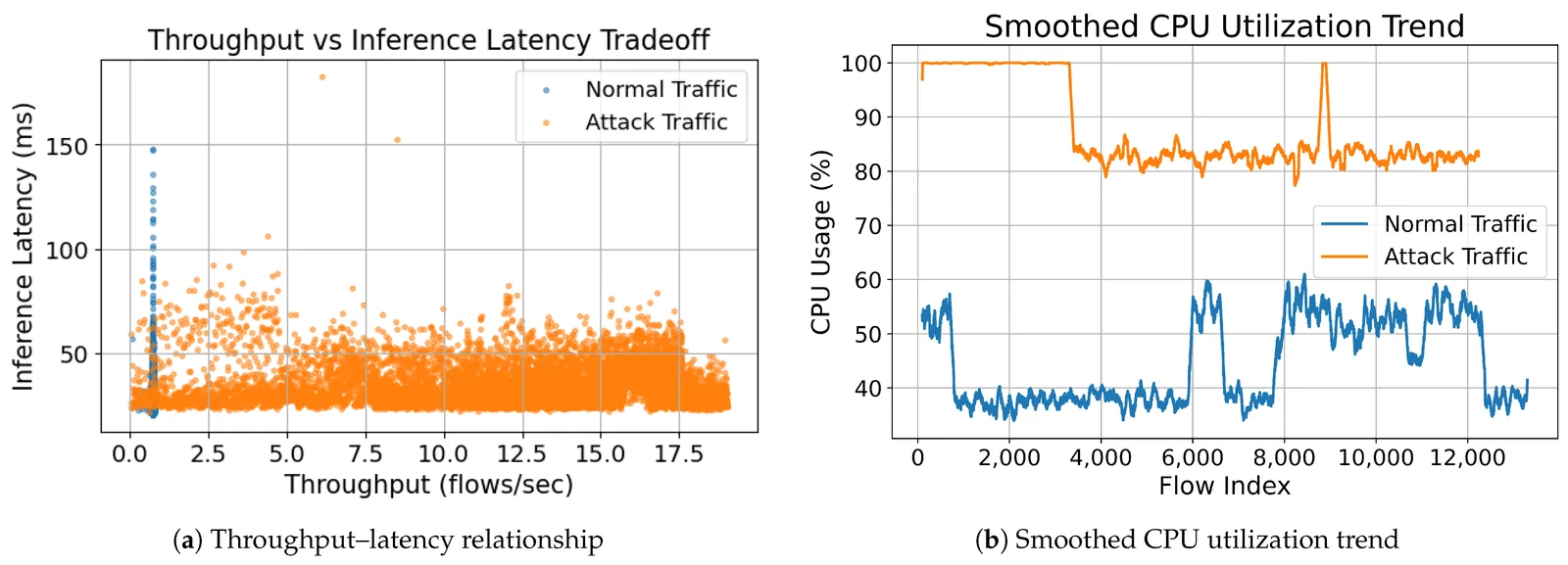
Real -time operational performance of the proposed IDS framework: (**a**) throughput–latency relationship under benign and adversarial traffic conditions; (**b**) smoothed CPU utilization trend showing stable runtime behavior during sustained traffic monitoring and attack generation.

To evaluate traffic-processing capability under varying workloads, throughput-versus-latency behavior is analyzed under both normal and malicious traffic. Figure 15a illustrates the relationship between processing throughput and inference latency over the deployment period. The experimental results indicate that normal traffic maintained relatively low throughput levels with tightly clustered latency. Under attack conditions, throughput increased substantially due to elevated malicious flow generation, reaching approximately 18 flows/s during peak attack intervals. Despite this increased workload intensity, the corresponding latency growth remained controlled rather than exponential.

The majority of attack-traffic inference operations remained below approximately 60ms even at elevated throughput, demonstrating that the framework maintains stable runtime responsiveness during high-volume traffic inspection. Although increased throughput introduced slightly higher latency variance, no severe processing bottlenecks or uncontrolled runtime degradation are observed. Therefore, within the evaluated single-switch, two-host SDN testbed, the framework maintained stable runtime responsiveness under increased malicious traffic. These results demonstrate the feasibility of the online inference pipeline at the tested scale, while validation in larger multi-switch and multi-controller SDN environments remains future work.

#### 6.4.4. CPU Utilization and Runtime Stability

To assess deployment sustainability, CPU utilization trends are continuously monitored during both normal and attack traffic experiments. Figure 15b presents the temporal CPU utilization of our IDS framework during runtime operation. Under benign traffic conditions, CPU utilization remained relatively stable at approximately 35–55%, reflecting efficient feature extraction and GRU inference during normal network activity. In contrast, attack traffic imposed substantially higher computational demand, with CPU utilization stabilizing near 80–85% due to intensified Zeek flow analysis, elevated packet processing, and accelerated inference operations triggered by high-volume malicious traffic.

Despite increased computational pressure, the framework maintained stable execution throughout the deployment period. The smoothed CPU utilization trend shows that the computational overhead remained bounded, rather than exhibiting unstable oscillations or uncontrolled resource escalation. Overall results indicate that the proposed framework can maintain stable runtime while supporting real-time SDN traffic monitoring, feature extraction, and GRU-based online inference.

#### 6.4.5. Comparative Runtime Discussion Under Benign and Adversarial Traffic

The comparative runtime analysis demonstrates that adversarial traffic increases inference latency, throughput demand, and CPU utilization compared with benign traffic. Median inference latency increased from approximately 24–28ms under normal traffic to approximately 32–38ms during adversarial conditions, while CPU utilization increased from approximately 35–55% to 80–85%. Throughput also increased during attack generation, reaching peak values near 18 flows/s.

Despite this increased workload, the runtime overhead remained bounded and operationally sustainable. The attack-traffic p95 inference latency remained approximately 52.19ms, indicating that 95% of attack-traffic inference operations are completed within nearly 52ms. Overall, the real-time deployment evaluation demonstrates that the proposed framework achieves a practical balance among detection effectiveness, runtime efficiency, and operational scalability. Since distributed key generation, encrypted aggregation, and threshold collaborative decryption are confined to the offline federated training stage, the deployed global IDS model performs low-latency online inference within the SDN environment, incurring no cryptographic overhead during runtime detection.

### 6.5. Detection-Performance Comparison with Existing Federated SDN-IDS Frameworks

Table 11 compares the detection performance and functional characteristics of the proposed framework with closely related recent federated IDS studies. The values for existing frameworks are taken from their respective publications and do not represent a controlled head-to-head evaluation, because the studies employ different datasets, binary or multiclass classification tasks, preprocessing procedures, learning architectures, client configurations, and hardware environments. Study [36] provides the closest privacy-preserving comparison through multi-key homomorphic encryption, whereas the FedLAD study [25] provides the closest comparison in terms of the evaluated datasets and real-time SDN validation. Study [37] provides explainability-oriented federated IDS comparisons. Therefore, Table 11 should be interpreted as a comparative positioning of the proposed framework rather than an absolute performance ranking. The real-time deployment of the proposed framework is evaluated using the global model trained on the InSDN dataset.

## 7. Discussion

The experimental results demonstrate that the proposed threshold-secured federated intrusion-detection framework achieves its main objectives: decentralized cryptographic security, privacy-preserving collaborative learning, explainable intrusion analysis, and practical SDN deployment. Compared with conventional HE-enabled federated IDS architectures, the framework strengthens cryptographic trust management while maintaining high detection performance and feasible runtime behavior under realistic SDN conditions.

### 7.1. Removal of Trusted Key Escrow and Distribution of Secret-Key Material

A major contribution of this work is removing the trusted key-generation dealer and centralized custody of a complete secret key commonly found in HE-enabled federated learning systems. In conventional architectures, including our previous TenSEAL-based framework [13], a trusted entity generates or stores a global secret key. Compromise of that entity may expose the encrypted aggregation process.

The proposed OpenFHE-based architecture instead uses multiparty distributed key generation so that participating clients retain separate threshold key material and no individual entity stores the complete secret key. Consequently, neither an individual client nor the aggregation server can decrypt ciphertexts using its own key material alone. Recovery of the aggregated global model requires partial decryption contributions from at least t clients. For the evaluated threshold configurations, the (3, 4), (5, 8), and (7, 12) settings tolerate at most 2, 4, and 6 colluding clients, respectively. These bounds follow directly from the condition *c* < *t*. Once the number of colluding clients reaches t, confidentiality of the aggregated ciphertext is no longer guaranteed. This property removes centralized key escrow and independent single-party decryption, but it does not make the complete SDN framework trustless. The aggregation server retains a coordination role during collaborative decryption, and the SDN controller remains trusted for runtime control-plane management. The contribution should therefore be understood as reducing centralized cryptographic trust in the federated-training subsystem rather than eliminating every trusted component or operational single point of failure.

### 7.2. Security–Performance Trade-Off

The cryptographic evaluation shows that threshold-secured federated learning introduces higher aggregation, communication, and decryption overhead than the previous TenSEAL-based implementation. This increase is expected because the proposed framework requires distributed key generation, multiparty coordination, partial-decryption-share generation, and collaborative decryption, which are absent from conventional single-key HE systems.

Despite this added complexity, the measured overhead remains feasible for the evaluated SDN scenarios. The results reveal a clear security–performance trade-off: additional computational and communication costs are incurred in exchange for distributed key generation, threshold decryption, and removal of centralized complete-secret-key custody. Importantly, these security mechanisms do not reduce detection effectiveness. The framework maintains high accuracy, precision, recall, F1-score, MCC, and ROC-AUC across the InSDN, CICDDoS2017, and CICDDoS2019 datasets.

The experiments with 4, 8, and 12 clients also show predictable increases in cryptographic overhead as the federation size grows. Although larger federations require more coordination and processing, encrypted aggregation remains reliable, and classification performance remains stable. These results indicate that the framework can support moderately scaled distributed SDN environments that require decentralized trust management.

### 7.3. Detection and Explainability Effectiveness

The proposed framework also extends our previous work by incorporating SHAP-based explainability. The results indicate that the federated GRU model relies on security-relevant traffic characteristics, such as abnormal packet-size distributions, transport-layer signaling anomalies, burst-oriented communication patterns, and irregular temporal flow behavior. Global and local SHAP analyses therefore provide evidence that the model’s predictions are associated with meaningful intrusion indicators rather than arbitrary correlations.

Explainability is especially important in SDN environments because automated responses, such as installing OpenFlow rules and blocking traffic, can directly affect legitimate network services. SHAP enables analysts to examine the reasoning behind IDS predictions before or alongside mitigation decisions. The fidelity evaluation further shows that removing the top-ranked SHAP features substantially reduces classification accuracy, confirming that these features meaningfully influence the model’s predictions. Thus, the framework improves both intrusion-detection effectiveness and operational transparency, supporting more trustworthy deployment of privacy-preserving IDS models in SDN environments.

### 7.4. Practical SDN Deployment Feasibility

The proposed framework is tested in a live SDN environment using Mininet, the Ryu controller, and Zeek-based monitoring. Unlike evaluations restricted to offline data or simulations, this deployment shows how the framework performs with real benign and malicious traffic. The results indicate consistent inference latency, maintained throughput, reasonable CPU usage, and dependable online classification.

A key architectural advantage is the separation of cryptographic training operations from the online inference pipeline. Distributed key generation, encrypted aggregation, and threshold decryption occur during federated training, while real-time deployment uses only the converged global IDS model. This separation reduces controller-side computational demand and prevents cryptographic latency from affecting real-time traffic classification. Overall, the findings demonstrate that threshold-secured federated learning can be integrated into an operational SDN environment while preserving decentralized security, high detection performance, explainability, and practical runtime efficiency.

The Ryu controller remains an operationally trusted component because it manages forwarding behaviour and control-plane decisions during runtime deployment. The proposed cryptographic framework does not protect against complete controller compromise. However, the controller does not participate in distributed key generation and does not hold client threshold key material; therefore, controller compromise does not directly reveal the distributed secret shares or previously encrypted local model updates.

### 7.5. Limitations and Future Work

Although the proposed framework demonstrates strong intrusion-detection performance, decentralized cryptographic security, explainability, and practical feasibility for SDN deployment, several limitations remain. The current framework focuses on binary intrusion detection with static federated participation and does not consider multiclass classification, dynamic participation, or highly non-IID data distributions. The present work also does not comprehensively evaluate robustness against sophisticated model-poisoning or adaptive malicious-client attacks. Furthermore, although the OpenFHE-based CKKS implementation exhibits stable empirical convergence, a formal analysis of how ciphertext approximation noise, threshold-decryption precision, and homomorphic approximation error affect convergence is beyond the scope of this study.

Additionally, the fidelity and cross-configuration stability analyses provide computational validation of the SHAP explanations; no formal user study involving professional security analysts is conducted. Future work will evaluate the usefulness of explanations, alert-triage effectiveness, analyst confidence, and incident-response time through expert assessment. Another limitation concerns collaborative decryption coordination. The current setup assumes that clients only submit partial decryptions for the legitimate aggregate of the prescribed federated round. A malicious coordinator might attempt to submit arbitrary or duplicate ciphertexts and leverage participating clients as decryption oracles. Future research will explore mechanisms for decryption-request authorization, transcript verification, and auditing. The SDN controller also remains trusted for runtime forwarding and control-plane operations; its compromise could affect operational availability and mitigation integrity, although it would not directly disclose distributed client key shares.

Finally, the scalability assessment is currently limited to 12 clients and is based on single runs for each configuration. Larger federations are likely to increase the overhead for encryption, aggregation, threshold decryption, and communication. Additionally, client heterogeneity and synchronization delays could impact convergence. Future research will involve testing with larger client groups using multiple runs with different random seeds. It will also explore optimized CKKS packing, parallel cryptographic processing, compression of model updates, hierarchical aggregation, methods resistant to poisoning attacks, and enhancing resilience against SDN-controller compromises.

## 8. Conclusions

This study presents a threshold-secured federated intrusion detection framework designed for SDN environments, combining distributed key generation, threshold homomorphic encryption, GRU-based federated learning, SHAP-based explainability, and real-time deployment validation. Testing on the InSDN, CICDDoS2017, and CICDDoS2019 datasets demonstrated the framework’s effectiveness in detecting intrusions, while supporting decentralized encrypted model updates and collaborative threshold decryption. The cryptographic analysis indicated that the extra aggregation, decryption, and communication costs provide a practical security–performance balance, eliminating the need for centralized complete-secret-key custody and enhancing protection against server-side decryption and collusion involving fewer than t clients.

Additionally, SHAP-based explainability enhanced transparency by pinpointing security-related traffic features linked to abnormal packet distributions, protocol-level anomalies, and burst attack behaviors. The real-time SDN setup using Mininet, the Ryu controller, and Zeek showed stable inference latency, consistent throughput, bounded CPU usage, and reliable detection during both normal and malicious traffic within the testbed. Quantitative results showed the framework achieved accuracy rates of 99.95%, 99.03%, and 99.65% on the InSDN, CICDDoS2017, and CICDDoS2019 datasets. During live SDN deployment, it reached 99.71% accuracy, 99.67% precision, 99.72% recall, and a 99.69% F1-score, with false-positive and false-negative rates of 0.31% and 0.28%, respectively. The 95th-percentile inference latency under attack traffic is approximately 52.19 ms. OpenFHE also reduced encryption latency by roughly three times compared to TenSEAL, though encrypted aggregation, collaborative decryption, and communication added some overhead. Overall, these results demonstrate that distributed key generation, encrypted federated aggregation, threshold collaborative decryption, explainable AI, and real-time SDN deployment can be combined into a single IDS framework, maintaining high detection accuracy and practical runtime efficiency at the tested scale.

## Figures and Tables

**Table 1:** Comparison of the proposed framework with existing SDN-Based IDS systems.

Ref.	Learning Paradigm	Core Technique	Secure Aggregation	Threshold Cryptography/DKG	XAI	Deployment	Remarks
[17,18,19,20,21,22]	Centralized	RNN, LSTM, GRU, GAN, ResNet	No	No	No	No	Requires pooling raw network data on a central server, severely compromising data privacy and scalability in multi-tenant SDNs.
[23]	Federated	DNN	No	No	No	No	Mitigates data centralization, but unencrypted plaintext model updates leave the system highly vulnerable to model inversion and gradient leakage attacks.
[24]	Federated	CNN–LSTM	No	No	No	No	Ensures update integrity but fails to provide cryptographic confidentiality during aggregation, exposing plaintext gradients to the central server.
[25]	Federated	XGBoost	No	No	No	Yes	Demonstrates SDN feasibility, yet depends on plaintext gradient exchange and provides no human-interpretable XAI.
[26]	Federated	Deep Learning	No	No	No	No	Susceptible to gradient leakage due to the absence of secure aggregation. Furthermore, its black-box nature hinders transparent security enforcement.
[27]	Federated	DNN	No	No	No	No	Uses transit encryption, but decryption at the central server exposes the model updates to an honest-but-curious aggregator.
[36]	Federated	1D-CNN–TransBiLSTM + HE	Yes	No	No	No	Protects model updates using HE but relies on a centralized trusted authority for key generation, creating a cryptographic SPOF.
[37]	Federated	MSDC-Net + XAI	No	No	Yes	No	Uses XAI to improve operational trust, but without secure aggregation, the federated pipeline remains exposed to security risks.
[38]	Federated	GNN and DL + XAI	No	No	Yes	No	GNN- and DL-based XAI improves transparency, but without secure aggregation, the federated pipeline remains mathematically vulnerable.
[39]	Federated	ANN + XAI	No	No	Yes	No	The ANN uses XAI for transparency, but without secure aggregation, the federated system remains vulnerable.
Previous Work [13]	Federated	GRU + HE (TenSEAL)	Yes	No	No	No	Secures model updates using standard HE but relies on a centralized trusted dealer for key generation, creating a SPOF, and operates as an offline black-box system.
Proposed	Federated	GRU + OpenFHE + SHAP	Yes	Yes (t-out-of-n)	Yes	Yes	removes centralized key-generation and complete-secret-key custody through distributed *t*-out-of-*n* threshold decryption, provides transparent threat interpretation using XAI, and validates real-time SDN inference/deployment.

**Table 2:** Threat model and security mitigation mechanisms.

Threat Entity	Adversarial Goal	Security Mechanism or Assumption
Honest-but-curious server	Infer local model updates or sensitive training information.	Threshold CKKS encryption and the absence of server-side secret-key ownership.
Colluding malicious clients	Reconstruct the global secret key or decrypt the aggregated ciphertext.	*t*-out-of-*n* distributed key generation and collaborative threshold decryption.
External eavesdropper	Intercept model updates during transmission.	End-to-end ciphertext communication using CKKS encryption.
Compromised participant	Infer sensitive training information or perform model-inversion attacks.	Decentralized encrypted aggregation and threshold cryptographic protection.
Communication adversary	Perform passive eavesdropping or traffic analysis.	Encrypted communication and a distributed trust architecture.

**Table 3:** Hardware and software environment.

Component	Specification
Processor	Intel Core i9 processor
GPU	NVIDIA RTX-series GPU
RAM	32 GB DDR4
Operating system	Ubuntu Linux 22.04
Programming languages	Python 3.10.12 and C++
Deep-learning framework	TensorFlow 2.13.0/Keras 2.13.1
Cryptographic library	OpenFHE
SDN emulator	Mininet
SDN controller	Ryu
Traffic monitoring tool	Zeek Network Security Monitor
Visualization libraries	Matplotlib 3.9.0 and SHAP 0.49.1

**Table 4:** Federated learning configuration.

Parameter	Value
Learning architecture	GRU-based IDS
Number of GRU layers	3
Hidden units	256, 128, and 64
Hidden-layer activation	ReLU
Output-layer activation	Sigmoid
Optimizer	Adam
Loss function	Binary cross-entropy
Number of federated rounds	15
Learning rate	0.0001
Local epochs per round	5
Dropout rate	0.3
Training/testing split	70%/30%
Number of clients	4, 8, and 12
Datasets	InSDN, CICDDoS2017, and CICDDoS2019

**Table 5:** CKKS cryptographic parameters.

Parameter	Value
Homomorphic encryption scheme	CKKS
Cryptographic library	OpenFHE
Ring dimension	8192
Batch size	4096
Multiplicative depth	2
Scaling-factor size	40 bits
Security level	128-bit security
Threshold scheme	(t,n) collaborative decryption
Threshold configurations	(3,4), (5,8), and (7,12)
Key-generation method	Distributed key generation (DKG)
Aggregation type	Encrypted federated aggregation

**Table 6:** Performance evaluation on different datasets.

Dataset	Accuracy (%)	Precision (%)	Recall (%)	F1 Score (%)	Global MCC (%)	Global Cohen’s Kappa	Global Log Loss
InSDN	99.95	99.92	99.98	99.95	99.90	0.999	0.25
CICDDoS2017	99.03	99.00	99.07	99.03	98.07	0.981	2.59
CICDDoS2019	99.65	99.47	99.91	99.69	99.29	0.993	0.78

**Table 7:** Controlled cryptographic comparison under the 12-client InSDN configuration.

Metric	Previous	Proposed
	TenSEAL Framework [13]	OpenFHE Framework
Encryption latency (s)	170.16	56.88
Aggregation latency (s)	25.07	97.71
Decryption latency (s)	3.11	88.99
Communication overhead (MB)	9615.67	14,093.67
Key-generation method	Centralized	Dealerless DKG
Decryption model	Single-key decryption	*t*-out-of-*n* collaborative decryption

**Table 8:** Top SHAP features and their security interpretations.

Rank	Feature	Mean SHAP	Security Interpretation
1	Init_Bwd_Win_Byts	0.091	Abnormal backward flow-control behavior associated with congestion-oriented attacks in SDN environments.
2	Pkt_Size_Avg	0.052	Irregular packet-size distributions generated by malicious network traffic.
3	Pkt_Len_Min	0.051	Malformed or asymmetric packet behavior commonly observed in attack traffic.
4	Bwd_Pkt_Len_Min	0.045	Abnormal reverse-channel communication patterns that may indicate suspicious traffic flows.
5	SYN_Flag_Cnt	0.040	TCP handshake anomalies and SYN-flooding behavior targeting SDN infrastructure.

**Table 9:** Stability of global SHAP explanations across federated client configurations.

Comparison	Spearman ρ	Common Top-10	Jaccard Similarity
4 vs. 8 clients	0.919	8	0.667
4 vs. 12 clients	0.905	8	0.667
8 vs. 12 clients	0.944	10	1.000

**Table 10:** Real-time detection performance during SDN deployment.

Model Evaluation	Accuracy (%)	Precision (%)	Recall (%)	F1-Score (%)	FPR (%)	FNR (%)
Real-time SDN IDS	99.71	99.67	99.72	99.69	0.31	0.28

**Table 11:** Performance comparison with closely related federated SDN-IDS frameworks.

Framework	Privacy Mechanism	Dataset	Accuracy (%)	F1-Score (%)	Explainability	Real-Time SDN
Ref. [25]	Standard FL	InSDN	98.07	98.50	No	Yes
Ref. [25]	Standard FL	CICDDoS2017	98.55	98.51	No	Yes
Ref. [25]	Standard FL	CICDDoS2019	98.38	98.90	No	Yes
Ref. [36]	Multi-key HE-FL	CICDDoS2019	99.92	99.96	No	No
Ref. [37]	Standard FL	InSDN	98.73	98.72	Yes	No
Proposed Framework	DKG + t-out-of-n CKKS	InSDN	99.95	99.95	Yes	Yes
Proposed Framework	DKG + t-out-of-n CKKS	CICDDoS2017	99.03	99.03	Yes	No
Proposed Framework	DKG + t-out-of-n CKKS	CICDDoS2019	99.65	99.69	Yes	No

## Data Availability

The InSDN, CICDDoS2017, and CICDDoS2019 datasets used in this study are publicly available from the sources cited in references [44,45,46]. The implementation associated with this study is available and is being progressively released at https://github.com/ictshamim09/threshold-fl-ids-sdn, (accessed on 17 August 2026). Initial experiment notebooks have already been deposited. The remaining implementation will be uploaded to the same repository under the MIT License before 31 August 2026.

## Abbreviations

The following abbreviations are used in this manuscript:

ANN	Artificial Neural Network
CDF	Cumulative Distribution Function
CKKS	Cheon–Kim–Kim–Song
DBN–LSTM	Deep Belief Network–Long Short-Term Memory
DDoS	Distributed Denial-of-Service
DKG	Distributed Key Generation
DNN	Deep Neural Network
FNR	False Negative Rate
FPR	False Positive Rate
GAN	Generative Adversarial Network
GNN	Graph Neural Network
GRU	Gated Recurrent Unit
IPFS	InterPlanetary File System
IoT	Internet of Things
LSTM	Long Short-Term Memory
NIDS	Network Intrusion Detection System
PCC	Pearson Correlation Coefficient
RNN	Recurrent Neural Network
SDN	Software-Defined Networking
SHAP	SHapley Additive exPlanations
SPOF	Single Point of Failure
XAI	Explainable Artificial Intelligence
